# Chemical Genetic Analysis and Functional Characterization of Staphylococcal Wall Teichoic Acid 2-Epimerases Reveals Unconventional Antibiotic Drug Targets

**DOI:** 10.1371/journal.ppat.1005585

**Published:** 2016-05-04

**Authors:** Paul A. Mann, Anna Müller, Kerstin A. Wolff, Thierry Fischmann, Hao Wang, Patricia Reed, Yan Hou, Wenjin Li, Christa E. Müller, Jianying Xiao, Nicholas Murgolo, Xinwei Sher, Todd Mayhood, Payal R. Sheth, Asra Mirza, Marc Labroli, Li Xiao, Mark McCoy, Charles J. Gill, Mariana G. Pinho, Tanja Schneider, Terry Roemer

**Affiliations:** 1 Merck Research Laboratories, Kenilworth New Jersey, United States of America; 2 Institute for Pharmaceutical Microbiology, University of Bonn, Bonn, Germany; 3 German Centre for Infection Research (DZIF), partner site Bonn-Cologne, Bonn, Germany; 4 Laboratory of Bacterial Cell Biology, Instituto de Tecnologia Química e Biológica, Universidade Nova de Lisboa, Oeiras, Portugal; 5 PharmaCenter Bonn, Pharmaceutical Institute, Pharmaceutical Chemistry, University of Bonn, Bonn, Germany; University of Tubingen, GERMANY

## Abstract

Here we describe a chemical biology strategy performed in *Staphylococcus aureus* and *Staphylococcus epidermidis* to identify MnaA, a 2-epimerase that we demonstrate interconverts UDP-GlcNAc and UDP-ManNAc to modulate substrate levels of TarO and TarA wall teichoic acid (WTA) biosynthesis enzymes. Genetic inactivation of *mnaA* results in complete loss of WTA and dramatic *in vitro* β-lactam hypersensitivity in methicillin-resistant *S*. *aureus* (MRSA) and *S*. *epidermidis* (MRSE). Likewise, the β-lactam antibiotic imipenem exhibits restored bactericidal activity against *mnaA* mutants *in vitro* and concomitant efficacy against 2-epimerase defective strains in a mouse thigh model of MRSA and MRSE infection. Interestingly, whereas MnaA serves as the sole 2-epimerase required for WTA biosynthesis in *S*. *epidermidis*, MnaA and Cap5P provide compensatory WTA functional roles in *S*. *aureus*. We also demonstrate that MnaA and other enzymes of WTA biosynthesis are required for biofilm formation in MRSA and MRSE. We further determine the 1.9Å crystal structure of *S*. *aureus* MnaA and identify critical residues for enzymatic dimerization, stability, and substrate binding. Finally, the natural product antibiotic tunicamycin is shown to physically bind MnaA and Cap5P and inhibit 2-epimerase activity, demonstrating that it inhibits a previously unanticipated step in WTA biosynthesis. In summary, MnaA serves as a new *Staphylococcal* antibiotic target with cognate inhibitors predicted to possess dual therapeutic benefit: as combination agents to restore β-lactam efficacy against MRSA and MRSE and as non-bioactive prophylactic agents to prevent *Staphylococcal* biofilm formation.

## Author Summary

*Staphylococcus aureus* and *Staphylococcus epidermidis* cause life-threatening infections that are commonly acquired in hospitals as well as the community and remain difficult to treat with current antibiotics. In part, this is due to the emergence of methicillin-resistant *S*. *aureus* and *S*. *epidermidis* (MRSA and MRSE), which exhibit broad resistance to β-lactams such as penicillin and other members of this important founding class of antibiotics. Compounding this problem, *Staphylococci* commonly colonize the surface of catheters and other medical devices, forming bacterial communities that are intrinsically resistant to antibiotics. Here we functionally characterize a family of 2-epimerases, named MnaA and Cap5P, that we demonstrate by genetic, biochemical, and X-ray crystallography means are essential for wall teichoic acid biosynthesis and that upon their genetic inactivation render methicillin-resistant *Staphylococci* unable to form biofilms as well as broadly hypersusceptible to β-lactam antibiotics both *in vitro* and in a host infection setting. WTA 2-epimerases therefore constitute a novel class of methicillin-resistant Staphylococcal drug targets.

## Introduction

*Staphylococcus aureus* is a leading cause of hospital and community-acquired infections by Gram-positive bacteria [1–3] and *Staphylococcus epidermidis* has emerged as the most common cause of biofilm infections on medical implant devices [4]. In large part, the difficulty in treating these infections lies in their broad resistance to β-lactams, an otherwise powerful class of antibiotics that include methicillin, penicillin, cephalosporins and carbapenems such as imipenem [5]. Mechanistically, β-lactams are bactericidal agents that lyse cells by inhibiting penicillin binding proteins (PBPs) involved in peptidoglycan (PG) synthesis and cross-linking in the cell wall [5, 6]. Methicillin-resistant strains of *S*. *aureus* (MRSA) and *S*. *epidermidis* (MRSE), however, have acquired an exogenous PBP (Pbp2a) that exhibits low binding affinity to β-lactams, thus rendering such strains clinically resistant to nearly all β-lactams [5, 7, 8]. Staphylococcal drug resistance is further exacerbated by the pathogen’s propensity to form a biofilm, in which many bacterial cells display a “persister”-like state of low metabolic activity and which renders antibiotics inactive, such as β-lactams that target active metabolic processes including growth and cell division [9, 10]. Biofilm formation also mediates antibiotic drug resistance by providing a complex and extensive polysaccharide extracellular matrix that serves as an effective physical barrier to antibiotic penetration into the cell [11–13].

**Fig 1:**
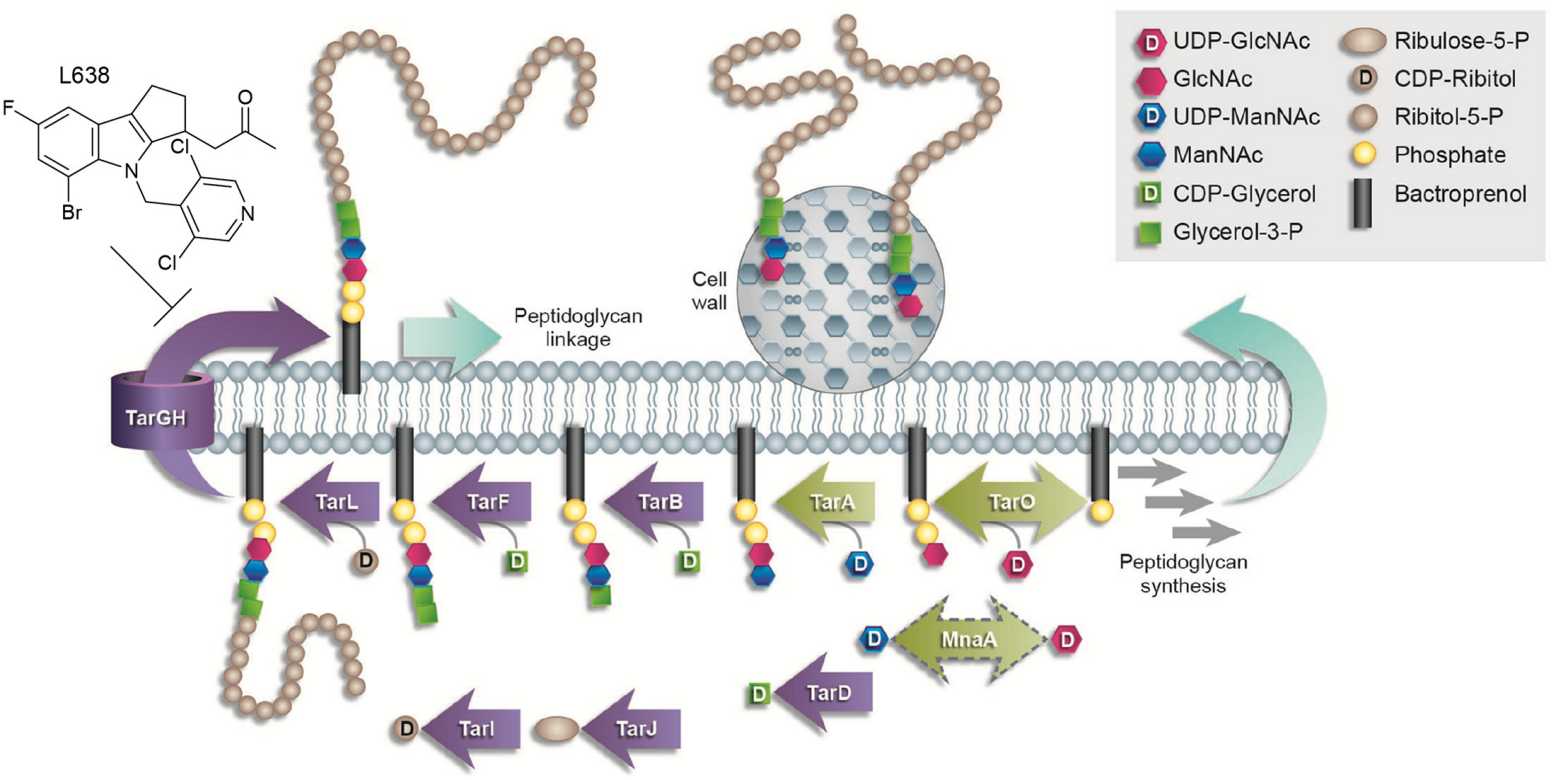
WTA biosynthesis pathway. WTA is sequentially synthesized by a series of Tar enzymes on a bactoprenyl phosphate carrier on the inner leaflet of the cell membrane and eventually transported to the outer leaflet where it is cross-linked to peptidoglycan. See inset for details. Non-essential early steps in WTA biosynthesis are shown as green arrows, late stage conditionally essential steps are shown as purple arrows. Note, MnaA is highlighted by a dashed bidirectional green arrow, highlighting its novel functional role as an epimerase that interconverts UDP-GlcNAc and UDP-ManNAc, thus providing substrates for TarO and TarA, respectively. L638 is a Staphylococcal-specific TarG inhibitor [33]. Schematic has been adapted from [34].

Wall teichoic acid (WTA) is an anionic glycophosphate cell wall polymer in Gram-positive bacteria that is present in roughly equal amounts to PG [14]. Interestingly, WTA has important functional roles in both the tolerance of methicillin-resistant *Staphylococci* to β-lactams [15–19] as well as in biofilm formation [20–24]. WTA is synthesized using the lipid carrier bactoprenyl phosphate and a sequential series of cytosolic-exposed plasma membrane associated Tar (*t*eichoic *a*cid *r*ibitol) enzymes, starting with TarO and TarA [19, 22, 25–27] (Fig 1). The polymer is subsequently translocated across the plasma membrane by an ABC transporter encoded by TarG and TarH [22, 28, 29] and ultimately cross-linked to the cell wall PG, upon which the liberated bactoprenyl carrier is recycled (Fig 1) [22, 25, 30–32].

Interestingly, genetic studies in *S*. *aureus* and *S*. *epidermidis* reveal that whereas deletions of early WTA biosynthetic enzymes are nonlethal, but cause diverse attenuated virulence phenotypes [27, 33, 35, 36], deletions of later steps in WTA biosynthesis are not generally tolerated and the enzymes are normally essential for growth [28, 37, 38]. This is referred to as an ‘essential gene paradox’, and may be explained either by 1) the accumulation of toxic WTA intermediates, or 2) sequestration of a non-recyclable pool of lipid carrier accumulating in late stage WTA deletion mutants such that bactoprenyl phosphate is unavailable to support PG biosynthesis (Fig 1) [19, 28, 37–40].

While WTA is dispensable for growth amongst Gram-positive bacteria [28, 35, 37, 38], it buffers methicillin-resistant *Staphylococci* from the action of β-lactam antibiotics [16, 17, 18, 33, 41] by coordinating peptidoglycan cross-linking [42] and targeting the major autolysin Atl [43]. Accordingly, genetic or chemical inhibition of Tar enzymes restores the susceptibility of MRSA and MRSE to β-lactams. Inhibitors to early (non-essential) enzymes in WTA biosynthesis are particularly appealing as non-bioactive adjuvants or combination agents that, paired with β-lactams, provide a promising strategy to treat MRSA and MRSE infections [16, 17, 33, 40, 41, 44, 45]. A growing number of small molecules targeting Tar enzymes have also been identified [16, 33, 34, 39, 41, 45, 46]. Perhaps best known is tunicamycin, a natural product structurally related to UDP-*N*-acetylglucosamine (UDP-GlcNAc), which inhibits TarO, the first enzyme in WTA biosynthesis [16, 47]. Tunicamycin demonstrates strong synergistic activity in combination with β-lactam antibiotics, presumably by depleting the buffering capacity WTA provides in β-lactam resistance of MRSA and MRSE. A variety of additional WTA inhibitors have also been demonstrated to target TarG, the membrane-associated subunit of the WTA transporter [33, 34, 40, 48].

WTA biosynthetic enzymes have been extensively characterized in *S*. *aureus* [19, 22, 25–27, 49, 50]. However, the identity and characterization of the 2-epimerase which interconverts UDP-GlcNAc and UDP-*N*-acetylmannosamine (UDP-ManNAc), each a substrate of TarO and TarA respectively (Fig 1), has remained largely restricted to *Bacillus subtilis* [51]. Two proteins, Cap5P and MnaA, share homology to the *B*. *subtilus* 2-epimerase [51] and have been suggested to potentially perform this function in *S*. *aureus* [52]. Cap5P and MnaA are 59.6% identical and 77.2% similar in their amino acid sequence, and each has been demonstrated to complement the phenotype of an *E*. *coli* strain lacking a 2-epimerase [52]. In addition, *S*. *aureus* Cap5P epimerizes ~10% of UDP-GlcNAc to UDP-ManNAc *in vitro*, which is comparable to the conversion levels observed for the *E*. *coli* and *B*. *subtilis* homologs [51, 52]. Disruption of *S*. *aureus cap5P*, however, did not yield an observable phenotype [52], implying that Cap5P and MnaA may share redundant functions associated with capsule and/or WTA biogenesis.

Herein we demonstrate that MnaA functions as the previously uncharacterized 2-epimerase that interconverts UDP-GlcNAc and UDP-ManNAc, thus providing the corresponding substrates of TarO and TarA in both *S*. *aureus* and *S*. *epidermidis*. Genetic evidence is provided demonstrating that MnaA is essential for WTA production and β-lactam resistance in MRSA and MRSE. Likewise, MnaA loss of function (LOF) mutants display restored susceptibility to β-lactam antibiotics in a mouse MRSA and MRSE thigh infection model. Whereas MnaA serves as the sole WTA 2-epimerase in *S*. *epidermidis*, MnaA and Cap5P provide overlapping roles in *S*. *aureus* WTA biosynthesis. We also demonstrate that MnaA is required for biofilm formation by methicillin-resistant *Staphylococci*, thus contributing to dual mechanisms of β-lactam resistance. We have determined the 1.9Å resolution crystal structure of *S*. *aureus* MnaA protein and describe critical residues for enzymatic dimerization, stability, and substrate binding. Finally, we demonstrate that tunicamycin, a known non-competitive inhibitor of TarO, also inhibits MnaA activity *in vitro* and discuss the potential therapeutic implications of WTA 2-epimerase inhibitors from the perspective of anti-Staphylococcal β-lactam combination agents.

## Results

### MnaA identification and functional role in WTA biogenesis in vivo and in vitro

Late steps of WTA biosynthesis are conditionally essential in *S*. *aureus* and *S*. *epidermidis*; genetic deletion or chemical inhibition of late WTA biosynthesis enzymes abolishes growth but can be tolerated provided early steps of WTA biosynthesis are also inactivated [28, 33, 37, 38, 40]. Accordingly, LOF mutations in early non-essential steps in WTA biosynthesis, such as TarO and TarA, act as bypass suppressors of late stage WTA inhibitors [16, 33, 34, 41]. To explore whether additional yet previously uncharacterized genes participate in early aspects of WTA biosynthesis, we used the previously published TarG inhibitor, L638 [33], as a chemical probe to screen for novel bypass suppressor mutations. Extensive L638-resistant (L638^R^) mutant selections were performed in both MRSA COL and MRSE CLB26329 strains.

**Fig 2:**
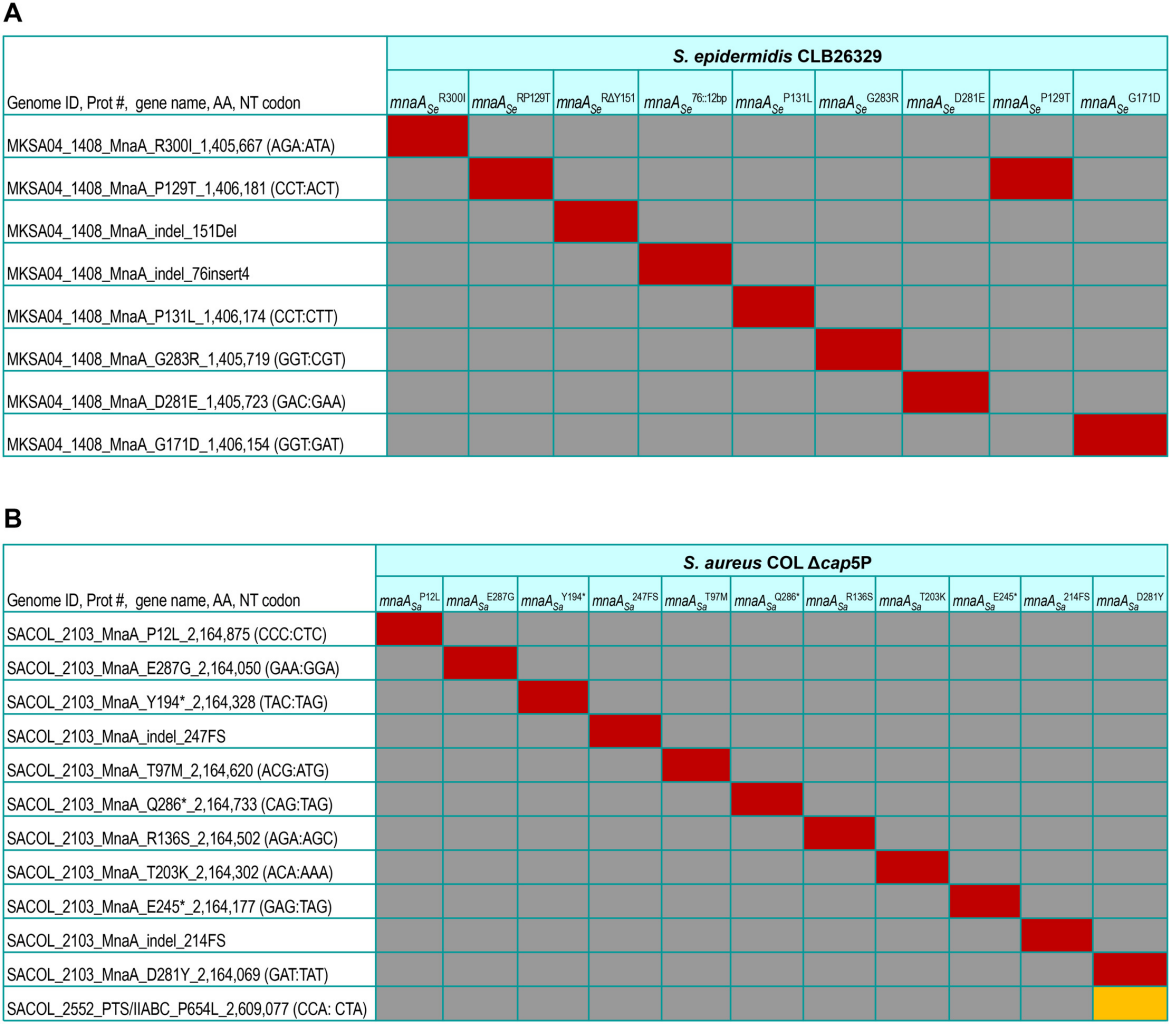
Whole-genome sequencing of L638^R^ mutants. Heat map summary of all non-synonymous mutations identified by Illumina-based whole-genome sequencing (100X genome coverage) of L638^R^ mutants in MRSE CLB26329 (A) or MRSA COL (B). Red, non-synonymous mutation; grey, no change versus parental genome sequence; yellow, non-synonymous mutations in genes other than *mnaA*. Genome position, base pair change, and resulting amino acid residue substitution are highlighted. Note: with only one exception (*Δcap5P mnaA*_*Sa*_^*D281Y*^), no additional non-synonymous mutations besides the indicated *mnaA* mutation were identified in each of the drug resistant strains examined.

As expected, multiple independently derived missense mutations mapping either to *tarG*, or LOF mutations mapping to *tarO* and *tarA* were identified in both strain backgrounds following whole genome sequencing (WGS) as previously reported [33]. Interestingly, in *S*. *epidermidis*, multiple (n = 9) independently derived L638^R^ mutations specifically isolated in this subsequent screen mapped to *mnaA*, encoding a putative UDP-GlcNAc:UDP-ManNAc 2-epimerase [52] not previously implicated as a suppressor of defects in late stage WTA biosynthesis in *Staphylococci* (Fig 2A). As WGS analysis indicates that each resistor isolate contains no additional non-synonymous mutations in their genome, we presumed *mnaA* mutations are causal for the L638^R^ phenotype observed. Unlike L638^R^ *tarG* mutations which are exclusively missense mutations conferring drug resistant amino acid substitutions to the target protein [33], L638^R^ *mnaA* mutations encompass nonsense, frameshift, and missense mutations (Fig 2A), therefore implying drug resistance is likely achieved by LOF mutations that possibly impair WTA biosynthesis. Finally, as TarO and TarA, respectively, require UDP-GlcNAc and UDP-ManNAc as substrates for initiating WTA polymer synthesis and an ortholog of MnaA was described to participate in *B*. *subtilis* WTA polymer synthesis [51] we investigated the functional role of MnaA in methicillin-resistant *Staphylococci*.

**Fig 3:**
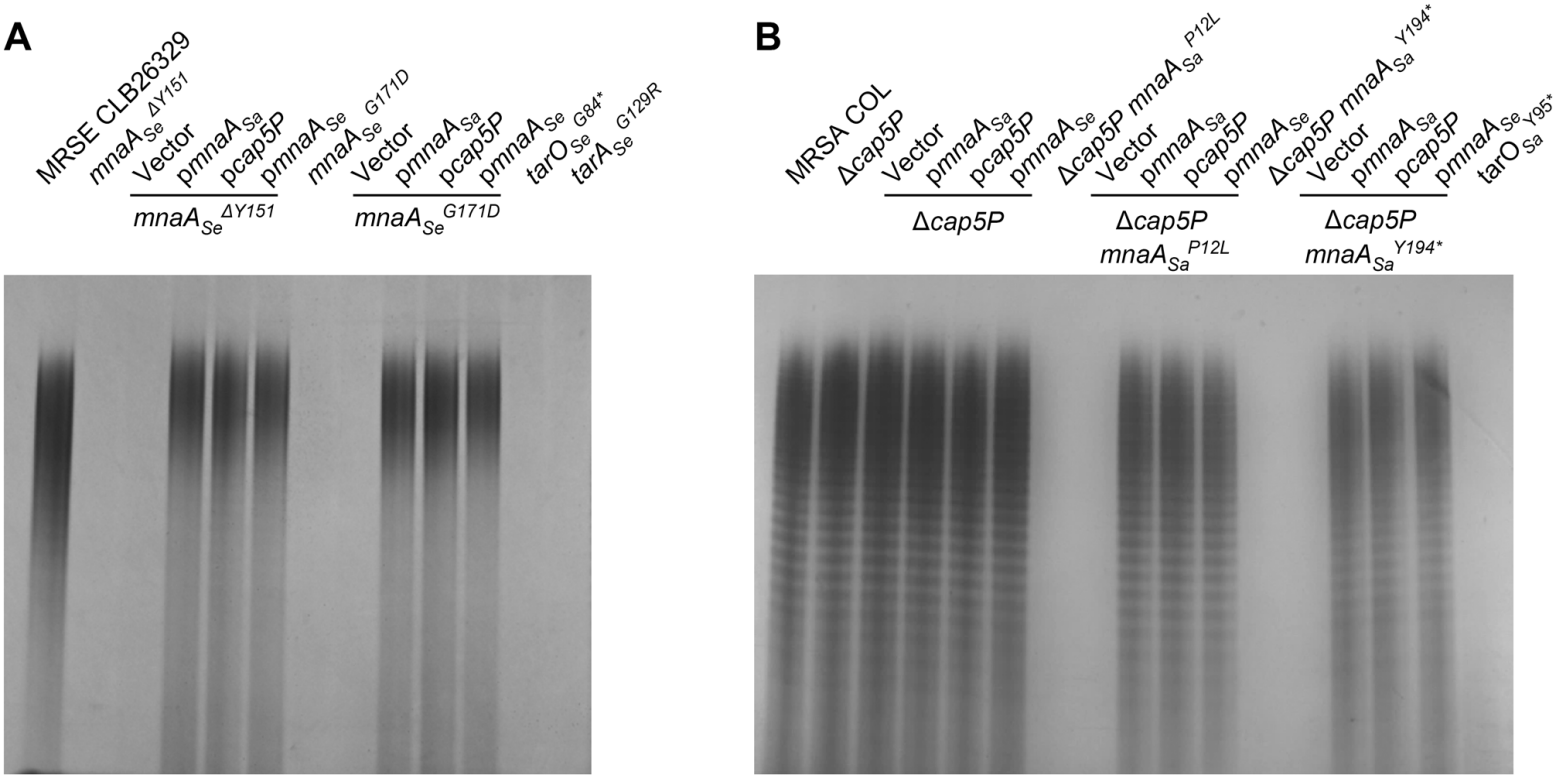
MnaA loss of function mutants in MRSA and MRSE fail to produce WTA. WTA extraction and SDS PAGE analysis from L638^R^ MRSE CLB26329 (A) and MRSA COL (B) mutants. Note, wild-type MRSA WTA polymers appear as a ladder of discretely sized bands whereas a more diffuse staining of MRSE WTA polymer is observed. WTA material was normalized to cell biomass prior to loading. Wild-type copies of *cap5P*, *mnaA*_*Sa*_, and *mnaA*_*Se*_, as well as the empty vector introduced into these strains for complementation studies are indicated. The *tarO* and *tarA* deletion mutants serve as a control for complete impairment of WTA polymer production.

To directly evaluate the consequence of these *mnaA* mutations, WTA of the corresponding mutants was extracted and polymer levels visualized on an alcian blue-silver stained SDS PAGE gel (Fig 3A and S1 Fig). As predicted, all MRSE *mnaA* mutants are completely depleted of WTA (Fig 3A and S1 Fig, right), mirroring *tarO*_*Se*_^*G84*^ * and *tarA*_*Se*_^*G129R*^ LOF mutants (Fig 3A; [33]). Importantly, like previously described MRSE *tarO* and *tarA* LOF mutants [33], all *mnaA* mutants display restored susceptibility to diverse β-lactams, with their minimal inhibitory concentration (MIC) below the clinical breakpoint defined for resistance to these agents (Table 1 and Table A in S1 Text). MRSE *mnaA* LOF mutants are up to 1000-fold more sensitive to imipenem, 256- to 512-fold more sensitive to nafcillin and 512-fold more sensitive to dicloxacillin compared to the parental MRSE strain (Table 1 and Table A in S1 Text). Notably, this dramatic antibiotic sensitization is specific to β-lactams (Table A in S1 Text).

**Table 1:** Genetic inactivation of *mnaA* in methicillin-resistant *Staphylococci* restores β-lactam susceptibility.

	MIC (μg ml^-1^)			
Strain	IPM	Naf	Dic	L638
MRSA COL	32	> 64	> 64	2
*Δcap5P*	32	> 64	> 64	2
*Δcap5P mnaA*_*Sa*_^*P12L*^	1	8	4	16
*Δcap5P mnaA*_*Sa*_^*P12L*^ */mnaA*_*Sa*_	8	32	32	2
*Δcap5P mnaA*_*Sa*_^*P12L*^ */cap5P*_*Sa*_	8	32	32	2
*Δcap5P mnaA*_*Sa*_^*P12L*^ */mnaA*_*Se*_	0.25	16	4	2
*Δcap5P mnaA*_*Sa*_^Y194^*	1	8	2	16
*Δcap5P mnaA*_*Sa*_^Y194^*/mnaA_*Sa*_	16	32	64	2
*Δcap5P mnaA*_*Sa*_^Y194^*/cap5P_*Sa*_	16	32	64	2
*Δcap5P mnaA*_*Sa*_^Y194^*/mnaA_*Se*_	4	16	16	2
MRSE CLB26329	64	> 64	> 64	4
*mnaA*_*Se*_^*ΔY151*^	0.25	0.25	0.125	16
*mnaA*_*Se*_^*ΔY151*^ */mnaA*_*Sa*_	> 64	64	> 64	8
*mnaA*_*Se*_^*ΔY151*^ */cap5P*	> 64	32	> 64	8
*mnaA*_*Se*_^*ΔY151*^ */mnaA*_*Se*_	> 64	32	> 64	8
*mnaA*_*Se*_^*G171D*^	0.25	0.125	0.125	16
*mnaA*_*Se*_^*G171D*^ */mnaA*_*Sa*_	> 64	32	> 64	8
*mnaA*_*Se*_^*G171D*^ */cap5P*	> 64	64	> 64	8
*mnaA*_*Se*_^*G171D*^ */mnaA*_*Se*_	> 64	64	> 64	8

Representative *mnaA* and *Δcap5P* mutations in MRSA and MRSE as well as complementation strains thereof are shown. Minimum inhibitory concentrations (MIC; μg ml^-1^) of β-lactams imipenem (IPM), nafcillin (Naf), and dicloxacillin (Dic) are provided. L638 is included to quantify drug resistance of bypass mutations.

Analogous L638^R^ mutant selections performed in MRSA COL were unsuccessful in identifying *mnaA* LOF mutants. Unlike *S*. *epidermidis*, however, *S*. *aureus* maintains a second 2-epimerase involved in serotype 5 capsular polysaccharide (CP5) synthesis, Cap5P (S2A Fig) [52]. To determine whether L638^R^ *mnaA* LOF mutants were not identified in MRSA COL due to a functional redundancy between Cap5P and MnaA, a *cap5P* deletion mutant was constructed (S3 Fig) and the L638^R^ studies were repeated. Under these conditions, in addition to identifying the expected *tarG* L638^R^ mutations as well as *tarO* and *tarA* LOF mutations, multiple (n = 11) independent resistor isolates obtained uniquely possess distinct mutations that map to *mnaA* and are predicted to inactivate gene function as well as directly confer L638^R^ drug resistance based on the absence of additional non-synonymous mutations in their genome following WGS analysis (Fig 2B). While MRSA COL *Δcap5P* exhibits no WTA depletion phenotype and remains resistant to β-lactams, MRSA COL *mnaA*, *Δcap5P* double mutants are completely devoid of WTA and are also highly sensitive to β-lactams (Fig 3B and Table 1 and Table A in S1 Text), again mirroring the restored β-lactam susceptibility of *tarO* and *tarA* deletion mutants [16, 33, 41]. Indeed, MRSA COL *mnaA Δcap5P* double mutants are 32- to 64-fold more sensitive to imipenem, 8- to 16-fold more sensitive to nafcillin, and 16- to 32-fold more sensitive to dicloxacillin compared to either *Δcap5P* or the isogenic parental strain (Table 1 and Table A in S1 Text).

Consistent with the functional role of MnaA in WTA biogenesis, MRSA COL *mnaA*, *cap5P* double mutants and MRSE CLB26329 *mnaA* single mutants display related growth and morphological defects as observed for *S*. *aureus ΔtarO* and *ΔtarA* mutants. For example, in both MRSA and MRSE strains examined, genetic inactivation of MnaA /Cap5P function led to a slightly reduced growth rate within the first 6 h of growth in fresh medium but no apparent difference in cell density versus the wild-type control over a 24 h extended growth period (S4 Fig). Similarly, super resolution microscopy analysis of MRSA COL *mnaA*, *cap5P* double mutants and MRSE *mnaA* single mutants revealed morphological phenotypes consistent with WTA depletion [16], including increased cell size heterogeneity and septation defects (S5 and S6 Figs).

Genetic complementation studies further demonstrate the overlapping functional activity of MnaA and Cap5P in *Staphylococci*. Complementing *Δcap5P mnaA*_*Sa*_^*P12L*^ and *Δcap5P mnaA*_*Sa*_^Y194^* with either *cap5P* or *mnaA*_*Sa*_ reintroduced on an inducible plasmid restored WTA polymer levels, resistance to each of the β-lactams tested, and wild-type sensitivity to L638 (Fig 3B and Table 1). Interestingly, cross complementation of these mutants with *mnaA*_*Se*_ also restored WTA production, albeit only partially restored wild-type drug susceptibilities (Fig 3B and Table 1). Similarly, *mnaA*_*Se*_^*Δ151*^ and *mnaA*_*Se*_^*G171D*^ were also fully complemented for each of the above phenotypes by reintroducing a wild-type plasmid-based copy of *mnaA*_*Se*_ (Fig 3A and Table 1). Strikingly, introduction of either *mnaA*_*Sa*_ or *cap5P* fully restored WTA production, β-lactam resistance, and L638 susceptibility of *mnaA*_*Se*_ LOF mutants (Fig 3A and Table 1). To further investigate β-lactam susceptibility phenotypes associated with *mnaA* inactivation, kill curve experiments were performed against MRSA and MRSE strains treated with the β-lactam imipenem. Whereas imipenem (4 μg ml^-1^) is ineffective in inhibiting growth of wild-type methicillin-resistant *Staphylococci*, imipenem displayed a dramatically restored bactericidal activity against MRSA *Δcap5P mnaA*_*Sa*_^*P12L*^ as well as MRSE *mnaA*_*Se*_^*Δ151*^ strains, leading to a 3 log reduction in viable cells within 7 hr of drug treatment (S7 Fig). Similar to other phenotypes examined, full complementation as well as heterologous complementation between *mnaA* orthologs were again observed (S7 Fig). Collectively, these data demonstrate that whereas MnaA seems to be one of two redundant UDP-GlcNAc:UDP-ManNAc 2-epimerases in MRSA COL, it is the sole 2-epimerase required for WTA biosynthesis in MRSE CLB26329.

**Fig 4:**
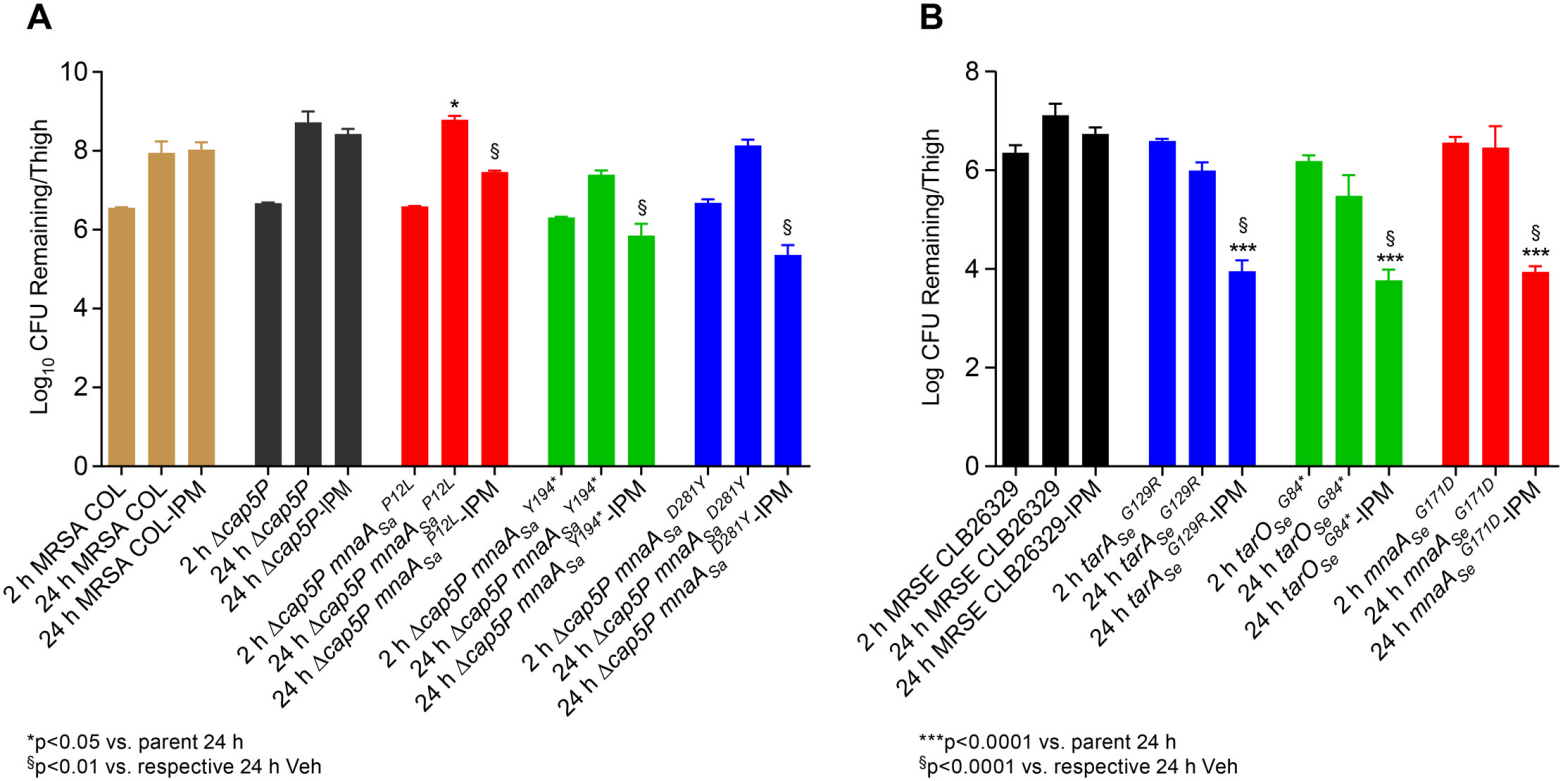
MRSA and MRSE MnaA LOF mutants are highly susceptible to imipenem in a murine thigh infection. Immune-suppressed CD-1 mice (5 per group) were challenged intramuscularly with the parental MRSA COL strain, MRSA *Δcap5P*, or MRSA *Δcap5P mnaA*_*Sa*_ LOF mutants (A) or with the parental MRSE strain versus *mnaA*_*Se*_, *tarO*_*Se*_ and *tarA*_*Se*_ LOF mutants (B) and treated three times daily (TID) with imipenem (IPM). Thighs were harvested at 24hrs, homogenized and plated to determine CFU per thigh. (A) Restored efficacy of IPM (10 mg kg^-1^) against MRSA *Δcap5P mnaA*_*Sa*_^*P12L*^, *Δcap5P mnaA*_*Sa*_^Y194^*, and *Δcap5P mnaA*_*Sa*_^*D281Y*^. Following IPM treatment, bacterial burden amongst mice infected with *Δcap5P mnaA*_*Sa*_^*P12L*^, *Δcap5P mnaA*_*Sa*_^Y194^*, and *Δcap5P mnaA*_*Sa*_^*D281Y*^ is reduced approximately 2–3 log at 24 hrs versus those infected with MRSA COL or *Δcap5P* controls. * p<0.01 versus parent at 24 hr; $ p<0.05 versus respective 24 hr vehicle. (B) Restored efficacy of IPM (2.5 mg kg^-1^) against MRSE *mnaA*, *tarO*, and *tarA* LOF mutants. Reduction in bacterial burden of mice infected with the *mnaA*_*Se*_^*G171D*^ is comparable to those infected with *tarO*_*Se*_^G84^* or *tarA*_*Se*_^*G129R*^ mutants, yielding an approximate 3 log reduction in 24 hr IPM treatment versus the wild-type control. Note, as MRSE CLB26329 is more susceptible to IPM than MRSA COL, its dose was reduced to 4-fold versus the MRSA efficacy study (A).

To evaluate the significance of the observed *in vitro* hypersensitivity of *mnaA*, *cap5P* double mutants to β-lactam antibiotics in an *in vivo* context, MRSA COL *Δcap5P mnaA*_*Sa*_^*P12L*^, *Δcap5P mnaA*_*Sa*_^Y194^*, and *Δcap5P mnaA*_*Sa*_^*D281Y*^ strains were used to conduct imipenem efficacy studies in a previously described murine deep thigh model of infection [53]. Imipenem is ineffective at treating animals infected with wild-type MRSA COL or the *Δcap5P* mutant when dosed three times daily (TID) with 10 mg kg^-1^ imipenem over 24 hours (Fig 4A) [33]. Conversely, imipenem efficacy is significantly restored against MRSA in *Δcap5P mnaA*_*Sa*_^*P12L*^, *Δcap5P mnaA*_*Sa*_^Y194^*, and *Δcap5P mnaA*_*Sa*_^*D281Y*^ mutants, ranging between a 2–3 log reduction of bacterial burden versus control strains after imipenem treatment (Fig 4A). As MRSE displays somewhat greater sensitivity to imipenem in our infection model, a lower dose (2.5 mg kg^-1^) was required to demonstrate restored efficacy of imipenem against the *mnaA*_*Se*_ mutant. Here again, mice administered imipenem (TID) at this non-efficacious dose and infected with the *mnaA*_*Se*_ mutant possessed a significantly reduced (> 3 log) bacterial burden versus the wild-type MRSE parent, similar to the effects of *tarO*_*Se*_^G84^* and *tarA*_*Se*_^*G129R*^ mutants (Fig 4B).

### MnaA-mediated WTA biosynthesis is required for biofilm formation

**Fig 5:**
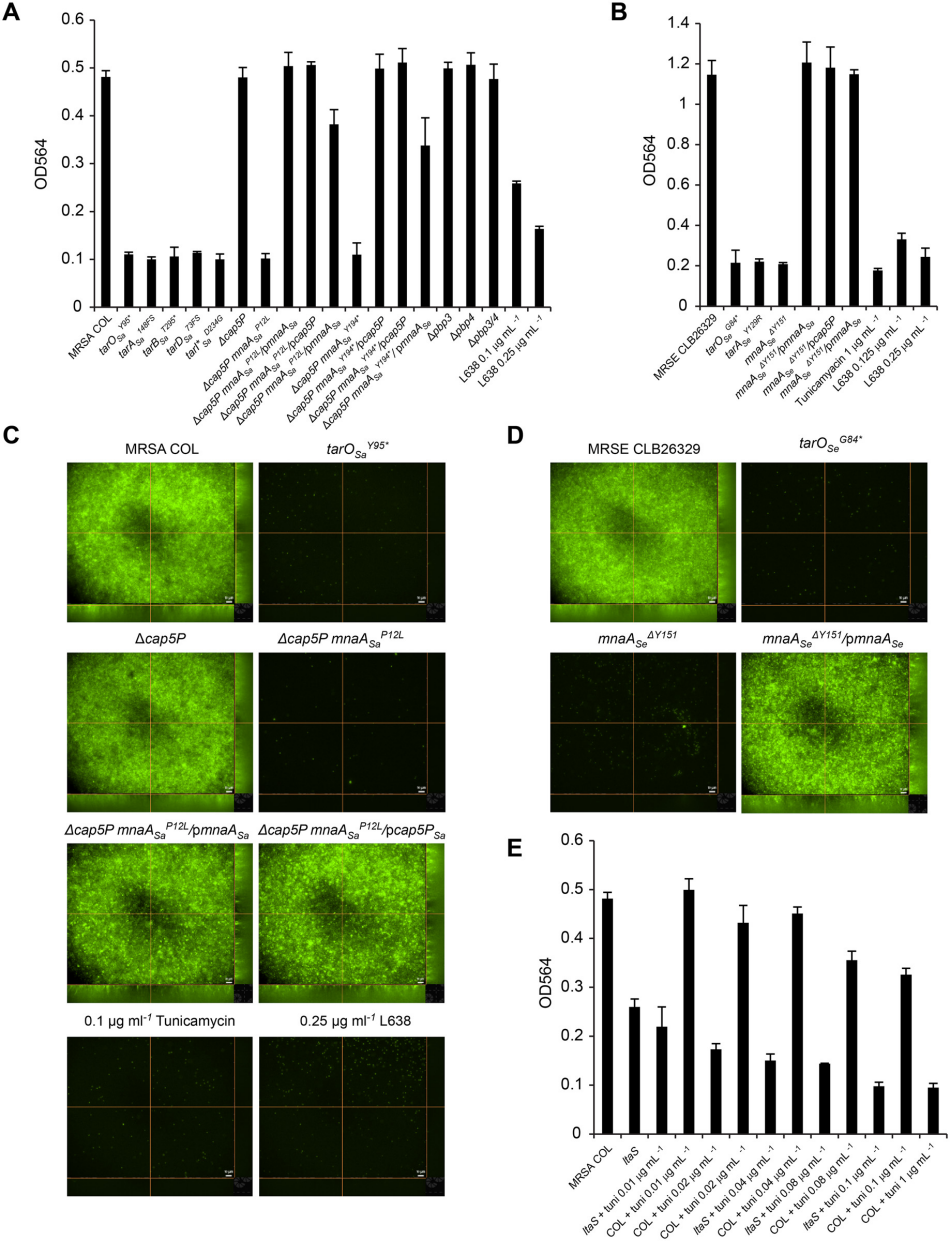
WTA is required for biofilm formation in methicillin resistant *Staphylococci*. For total biofilm quantification, biofilms were grown in triplicates for 24 hours in 96-well plates with or without indicated sub-MIC concentrations of WTA inhibitors for MRSA COL (A,C,E) and MRSE CLB26329 (B,D) strains. Genetic complementation of described mutants was performed using plasmid-based copies of wild-type *cap5P* (p*cap5P*), *mnaA*_*Sa*_ (p*mnaA*_*Sa*_), and *mnaA*_*Se*_ (p*mnaA*_*Se*_) as indicated. Biofilms were stained with safranin and dissolved in glacial acetic acid before OD_564_ was measured. Bars represent mean OD, error bars represent standard deviation. For Epi fluorescence microscopy, biofilms of MRSA (C) and MRSE (D) were grown as above in black clear bottom plates and stained with *Bac*Light Green fluorescent stain. Z-stacks were obtained at 60x magnification. Scale bar = 10 μm.

Since deletion of *tarO* has been shown to be important for biofilm formation and attachment [20–24], we evaluated the role of *mnaA*, *cap5P*, and other WTA biosynthesis genes in this process. MRSA COL strains with LOF in early (*tarA*, *tarO*) and intermediate (*tarB*, *tarD*, *tarI’*) steps in WTA biosynthesis [33] were all substantially defective in biofilm formation (Fig 5A). Conversely, *Δpbp3* and *Δpbp4* single mutants as well as the *Δpbp3 Δpbp4* double mutant control strains faithfully produced biofilms indistinguishable from the wild-type MRSA COL parent (Fig 5A). MRSE strains deleted of *tarO*_*Se*_^G84^* or *tarA*_*Se*_^*G129R*^ also failed to form robust biofilms (Fig 5B). Paralleling this WTA-mediated role in biofilm formation and attachment, *mnaA*_*Se*_ mutants and *Δcap5P mnaA*_*Sa*_ mutants similarly displayed impaired biofilm formation. MRSA COL *Δcap5P* mutants, however, failed to impair biofilm formation (Fig 5A), consistent with its lack of phenotypes related to WTA biogenesis, β-lactam susceptibility, and virulence. Fluorescence microscopy on stained, similarly grown, and treated biofilms confirmed these phenotypes (Fig 5C and 5D and S8 and S9 Figs). Genetic complementation of the biofilm impairment observed in *Δcap5P mnaA*_*Sa*_^*P12L*^ *and Δcap5P mnaA*_*Sa*_^Y194^* is fully achieved by reintroducing either wild-type *S*. *aureus* gene and partially achieved by *S*. *epidermidis mnaA* (Fig 5A and 5C and S8 Fig). Similarly, impaired biofilm formation of *mnaA*_*Se*_^*ΔY151*^ was faithfully complemented by reintroduction of *mnaA* as well as *S*. *aureus mnaA* or *cap5P* (Fig 5B and 5D and S9 Fig), again reiterating a strong functional overlap between these 2-epimerases.

To test whether known inhibitors of WTA biogenesis similarly disrupt biofilm formation, MRSA and MRSE strains were grown as above and treated with sub-MIC concentrations of tunicamycin or L638. Tunicamycin treatment at levels shown to completely inhibit WTA production [17, 35] decreased biofilm formation to amounts similar to those achieved by genetic inactivation of its target, TarO (Fig 5B, 5C and 5E and S8 and S9 Figs). Similarly, L638 treatment at sub-MIC levels that do not dramatically affect growth produce a dose-dependent inhibition of biofilm formation (Fig 5A, 5B and 5C and S8 and S9 Figs). Conversely, neither tunicamycin nor L638 similarly tested singly or in combination with a sub-MIC level of imipenem significantly disrupted the gross morphology, adherence, viability or antibiotic susceptibility of pre-existing biofilms (S10 Fig). Therefore, inhibition of WTA synthesis can prevent the establishment of a biofilm growth state, presumably by disrupting the early attachment step in biofilm colonization, but does not significantly impair biofilm viability or disrupt the extracellular matrix of pre-existing biofilms.

Lipoteichoic acid (LTA), another cell surface teichoic acid common to Gram-positive bacteria, has also been reported to play a role in biofilm formation [54, 55] and co-depletion of WTA and LTA demonstrate a synthetic lethal genetic interaction in both *B*. *subtilis* [56] and *S*. *aureus* [57]. Accordingly, we tested whether depletion of both WTA and LTA synergistically impair biofilm formation. Since LTA is essential [58], a previously described partial LOF *ltaS*_*Sa*_ mutant that produces lower levels of LTA than the parental strain [59] was tested for biofilm formation both in the absence and presence of increasing tunicamycin concentrations. Whereas the *ltaS* defective strain exhibits a slight 2-fold reduction in biofilm formation, treatment with sub-MIC levels of tunicamycin produces a dose dependent further reduction in biofilm formation approaching that of *tar*_*Sa*_ mutants (Fig 5E). Interestingly, this baseline level of residual biofilm formation in the *ltaS*_*Sa*_ mutant background was achieved with ~10 percent the normal concentration of tunicamcyin required to similarly impair biofilm in the wild-type parent strain (Fig 5E). Such an apparent synergistic effect further suggests a functional interdependence between these teichoic acid biosynthetic pathways and biofilm formation.

### MnaA crystal structure and binding to tunicamycin

**Fig 6:**
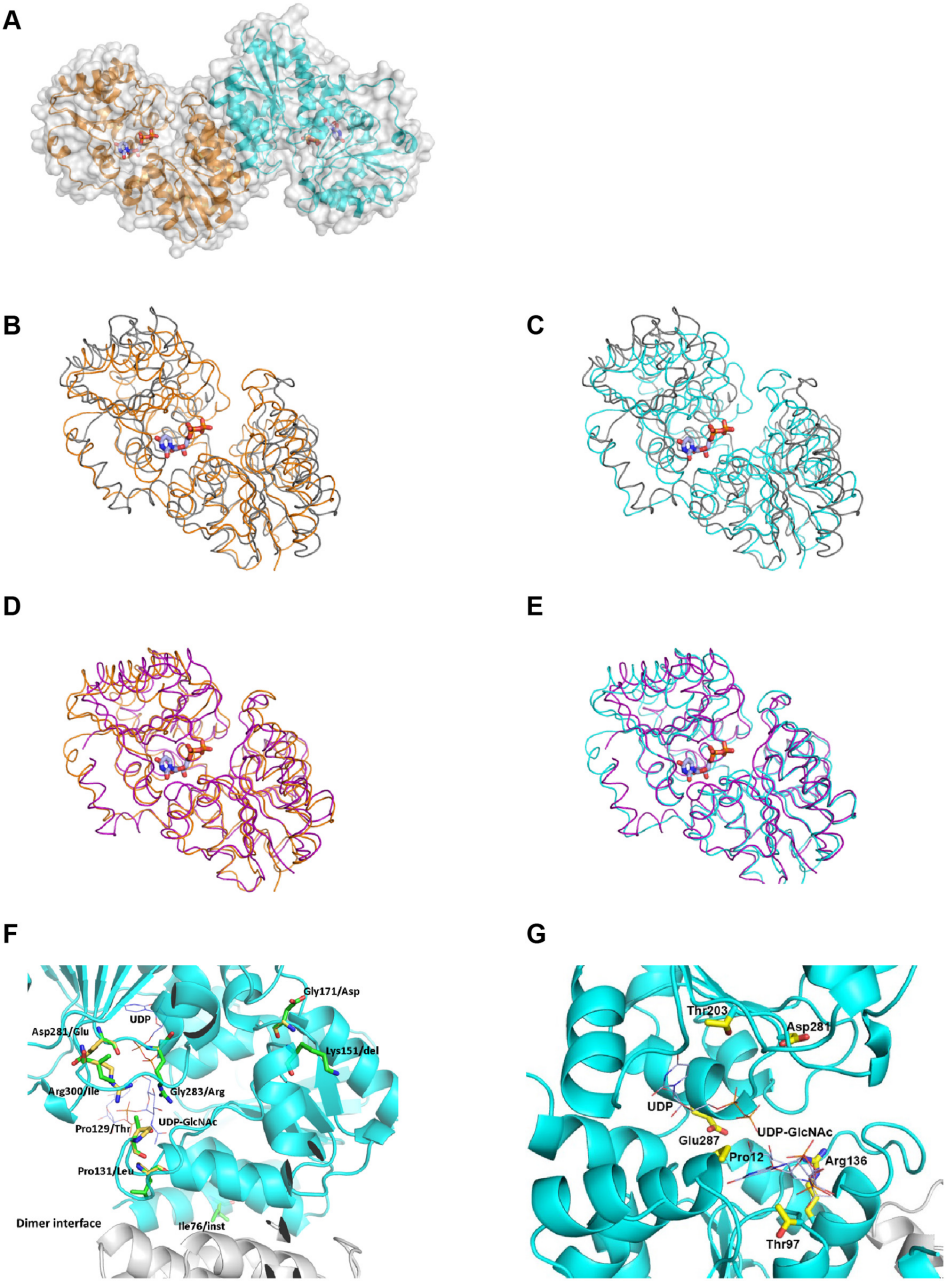
Mapping of MnaA LOF mutations into the MnaA crystal structure reveal key residues for substrate binding site stability and charge. (A) Overall MRSA COL MnaA crystal structure. The molecular surface is shown in grey. The protein is represented as a cartoon. In all figures one monomer is consistently colored in orange and the other in cyan, and the bound UDP molecules are shown as sticks, methyl groups colored in light blue. Nitrogen, oxygen and phosphor atoms are in blue, red or orange, respectively. (B,C,D,E) Comparison with the *M*. *jannaschii* structure in “opened” form (PDB 3NEQ) or “closed” form (PDB 3NES). Both structures are represented as ribbons, one monomer at a time, and UDP as sticks. (B) and (C) compares the opened form, in grey, with each monomer, while the superposition is with the closed form, in (D) and (E), drawn in purple. The RMS deviation in Cα positions are 1.6Å for 262 atoms, 1.6Å for 256 atoms, 1.5Å for 321 atoms, and 1.3Å for 336 atoms, for the superpositions in cartoon (B), (C), (D) and (E), respectively. (F) Mapping MRSE LOF mutants. Eight mutation sites are mapped onto the X-ray crystal structure of UDP bound MRSA COL MnaA. The allosteric site ligand UDP-GlcNAc was taken from the structure of UDP-GlcNAc bound *B*. *anthracis* 2-epimerase (PDB ID 3BEO). UDP and UDP-GlcNAc are displayed as thin lines with the carbon atoms colored in light blue. One monomer of MnaA dimer is colored in cyan and the other in white. The mutation sites are highlighted in stick. The carbon atoms of the wild-type residues are colored in yellow; those of the mutant residues are in green. (G) Mapping MRSA *mnaA* LOF mutants. LOF mutations isolated in MRSA COL MnaA are highlighted. All coloring as in (C), but for simplicity, only the original sequence is shown.

The MRSA COL MnaA crystal structure was solved at 1.9Å resolution (Fig 6A). The protein crystallizes with a dimer in the asymmetric unit. The structure is closely related to that of other bacterial 2-epimerases (root-mean-square (RMS) deviation differences with *E*. *coli* MnaA in Cα positions for all atoms in a monomer between 1.40 and 2.90Å depending on the chains being compared), and a similar dimerization interface and quaternary structure is observed (Fig 6B). While there are significant structural differences between the *E*. *coli* and *S*. *aureus* models, they are located on the protein surface, away from either the substrate binding site or the oligomerization interface. Surprisingly, there are differences between the two monomers in the *S*. *aureus* MnaA structure: the RMS. deviation in positions for all Cα is about 1.0Å. We refer in the following the structure of the ternary complex between MnaA, UDP and UDP-GlcNAc as the “closed” form [60], and other states of the protein, either in apo form or a binary complex with UDP, as the “opened” form, consistent with past structural characterization of the enzymes [60]. This difference between monomers is comparable to the differences with the structure of the *E*. *coli* enzyme (PDB entry 1F6D) in opened form, 1.0 to 1.9Å, depending on which chains are compared. However, further comparisons show that the differences with the closed form are less when comparing to one of the monomers in the *S*. *aureus* crystal structure rather than the other (RMS deviation of 1.2Å for 354 atoms in the superposition with *B*. *anthracis* MnaA in complex with UDP and UDP-GlcNAc (closed form), PDB entry 3BOV, versus 1.4Å for 340 atoms for the other monomer (See also Fig 6)). In addition to differences in the quaternary structure, other significant local structural rearrangements distinguish the two monomers. Notably, His 205 to Gly 211 (*E*. *coli* His 213 to Gly 219, *B*. *anthracis* His 209 to Gly 215) differ significantly, but in the monomer nearer to the closed form adopts a conformer similar to the one observed in the closed form [61]. By contrast, the same loop in the other monomer has a local fold similar to the one found in the *M*. *jannaschii* epimerase in apo form (PDB entry 3NEQ). Collectively, crystallographic results described here allow for a more refined understanding of the enzyme regulation at a structural level: a dynamic equilibrium between the opened form and an “intermediate closed” conformer of the enzyme is present in solution in apo form or in presence of UDP only. The equilibrium is moved and locked towards the “closed” form in the ternary complex with UDP—UDP-GlcNAc.

L638^R^ bypass mutants corresponding to MnaA LOF mutants were mapped to the MRSA COL MnaA crystal structure (Fig 6C and 6D). Among eight MnaA LOF mutations isolated in MRSE (Fig 2A), only the Gly283/Arg and Pro131/Leu mutations are located at the ligand (UDP and UDP-GlcNAc) binding sites. Gly283/Arg is positioned at an area across both the UDP and UDP-GlcNAc binding sites (UDP-GlcNAc binding site was mapped from the structure of UDP-GlcNAc bound *Bacillus anthracis* 2-epimerase (PDB ID 3BEO) through structure overlay) as shown in the X-ray structure of MnaA (Fig 6B). The large side-chain of Arg residue may cause van der Waals (VDW) clashes with the ligands and surrounding residues, thus interfere with binding of substrate UDP-GlcNAc and intermediate UDP, and destabilize the protein. The Pro131/Leu mutation is adjacent to the UDP-GlcNAc binding site and close to the dimer interface; it could both affect substrate binding and dimer stability through VDW conflicts by the Leu side-chain. Mutations Pro129/Thr, Gly171/Asp, Asp281/Glu and Arg300/Ile introduce amino acids with bulkier side-chains, compromise the favorable hydrogen bonds and hydrophobic interactions around the wild-type residues, and thus decrease protein stability. Protein stability could also be dramatically reduced by the deletion at Lys151 and the insertion at His76, the latter of which is located right in the middle of a helix, which is packed against the other monomer at the dimer interface. Mapping of MnaA LOF mutations isolated in MRSA COL *Δcap5P* (Fig 2B) into the *S*. *aureus* MnaA structure shows that all the sequence changes are in close proximity to the substrate binding site or the UDP-GlcNAc binding site (Fig 6D), therefore rendering the enzyme inactive.

**Fig 7:**
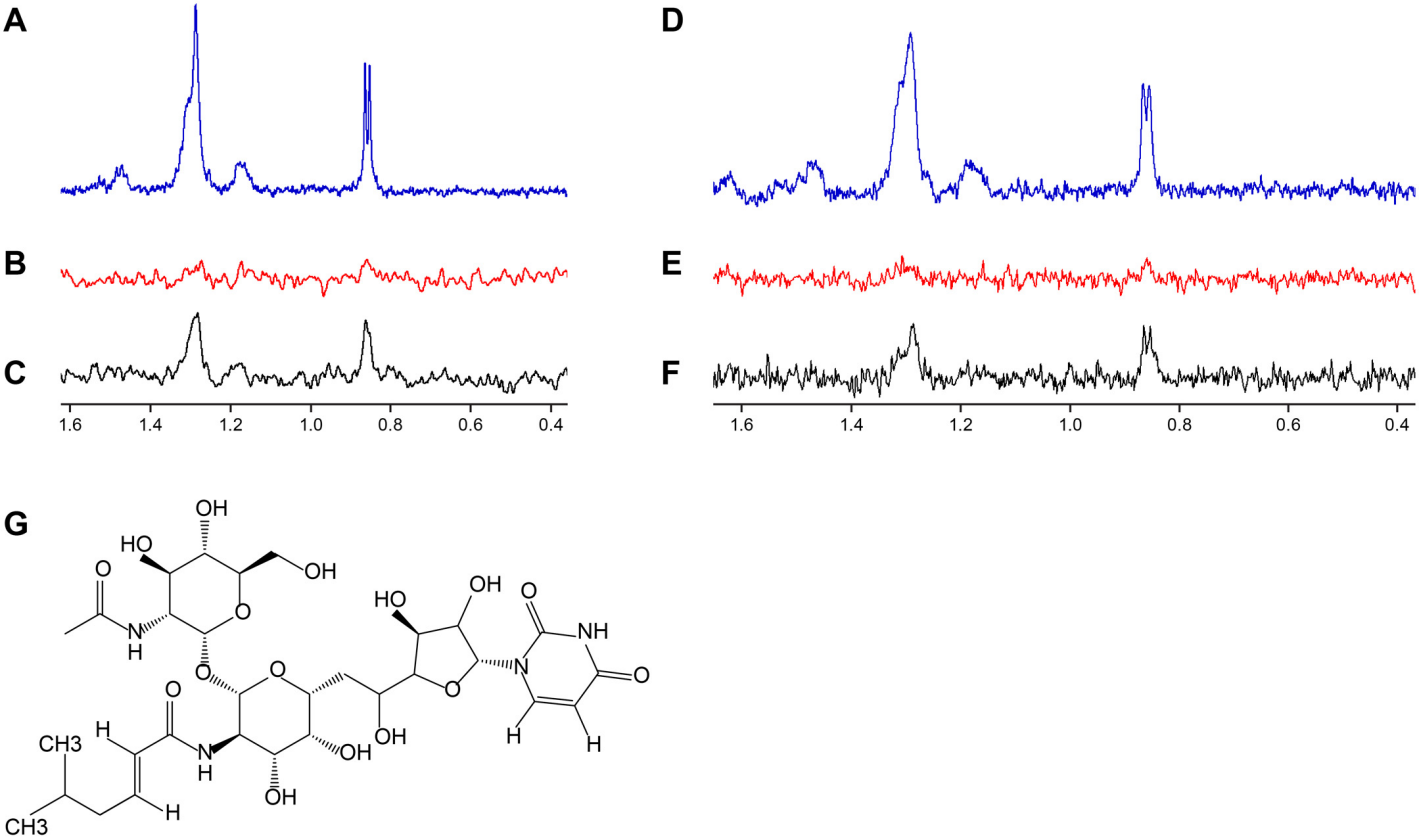
Biophysical studies demonstrate MnaA and Cap5P bind tunicamycin. (A, D) 600 MHz 1H NMR spectra of 15 μM tunicamycin. (B, E) 1H NMR STD spectra of 15 μM tunicamycin without 2-epimerase. (C) 1H NMR STD spectra of 15 μM tunicamycin in presence of 5 μM MnaA. (F) 1H NMR STD spectra of 15 μM tunicamycin in presence of 5 μM Cap5P. Saturation of the protein was achieved with a Gaussian pulse cascade resulting in a total saturation time of 3s. The protein resonances were saturated at 100 Hz and the off resonance was set to -120 ppm. Tunicamycin-specific peaks in NMR STD spectra were only obtained in the presence of MnaA or Cap5P. (G) Structure of tunicamycin.

Tunicamycin targets multiple UDP-GlcNAc binding enzymes. At higher drug concentrations tunicamycin binds MraY, a UDP-N-acetylmuramoyl-pentapeptide: undecaprenyl-phosphate phospho-N-acetylmuramoyl-pentapeptide transferase enzyme involved in peptidoglycan synthesis [62]. However, at low drug concentrations, tunicamycin selectively inhibits TarO [34, 47]. Considering MnaA and Cap5P are epimerases responsible for interconverting UDP-GlcNAc and UDP-ManNAc and that TarO utilizes the same substrate, we tested whether tunicamycin may also bind MnaA and Cap5P. To test this possibility, we performed saturation transfer difference (STD) nuclear magnetic resonance (NMR) studies, which allow for the detection of transient binding of small molecules to proteins [63]. Such studies using 15 μM tunicamycin in the presence or absence of 5 μM of *S*. *aureus* MnaA or Cap5P protein revealed binding of tunicamycin to both MnaA and Cap5P, as evidenced by the tunicamycin specific peaks appearing only when run in the presence of 2-epimerases (Fig 7A, 7C, 7D and 7F).

### Tunicamycin inhibits MnaA enzymatic activity

**Fig 8:**
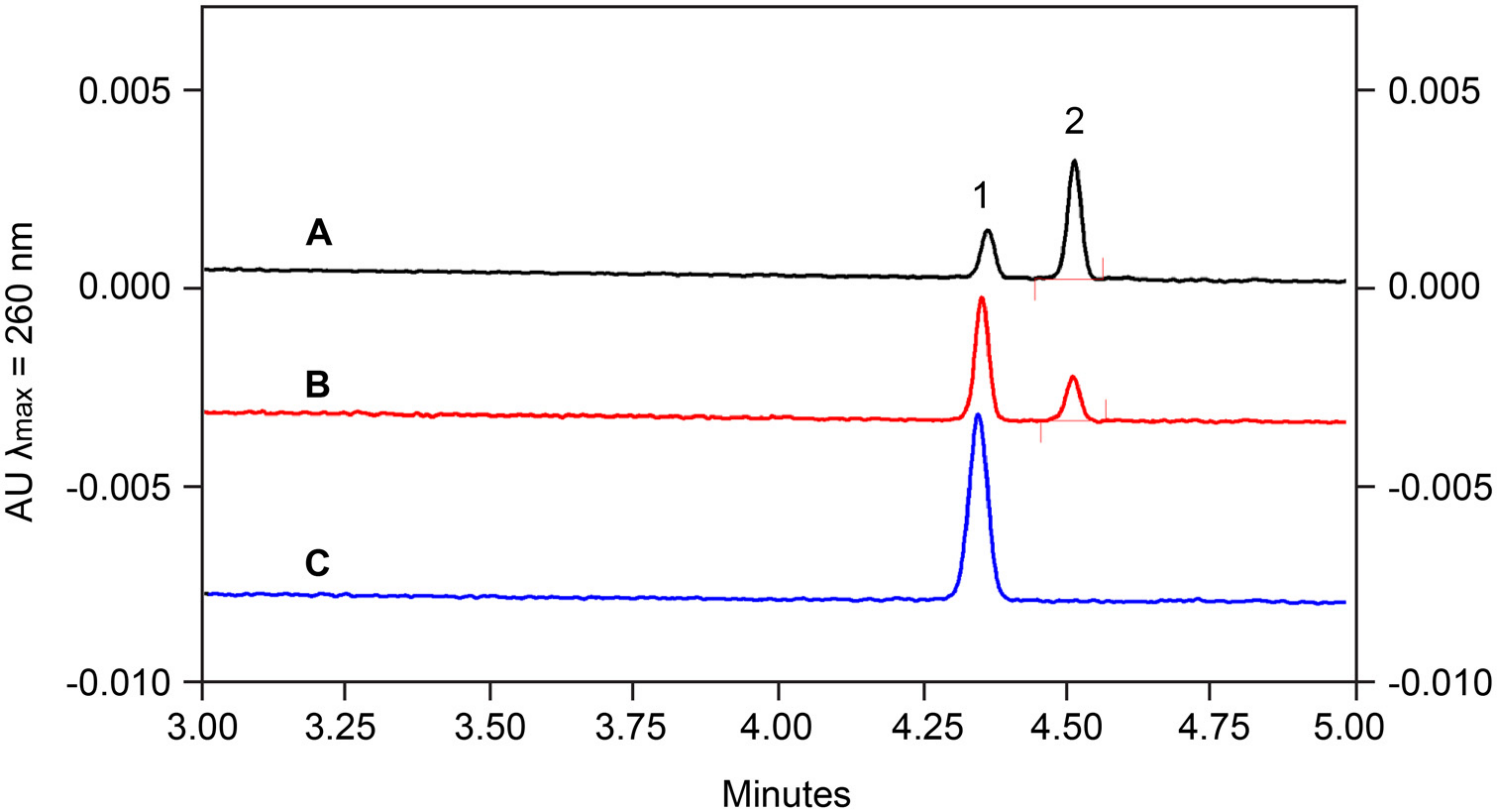
Tunicamycin inhibits MnaA in a concentration-dependent manner. Representative electropherograms of the MnaA-catalyzed conversion of UDP-ManNAc to UDP-GlcNAc in the absence of tunicamycin (A), in the presence of 100 μM (B) and 200 μM tunicamycin (C). Substrate concentration is 100 μM. Peaks: **1** UDP-ManNAc; **2** UDP-GlcNAc.

Binding of tunicamycin indicated that MnaA may represent an additional target of the nucleoside antibiotic in WTA biosynthesis beyond TarO. Functional reconstitution of the MnaA-catalyzed reaction *in vitro* followed by capillary electrophoresis (CE) analysis with UV detection showed the interconversion of UDP-GlcNAc and UDP-ManNAc, confirming 2-epimerase activity (S11 Fig). Enzyme kinetics (Michaelis-Menten constant, K_m_, and maximal velocity, V_max_) were determined for both, forward and reverse reaction. The conversion of UDP-GlcNAc to UDP-ManNAc (forward reaction) was in the linear range (steady-state phase) for up to 75 min (S12A Fig), while the reverse reaction exhibited a lag-period of 50 min after reaction initiation and reached equilibrium after 180 min (S12B Fig). MnaA displayed a K_m_ value of 411 ± 57 μM for UDP-GlcNAc and a V_max_ value of 0.171 ± 0.037 μmol/min/mg protein. A K_m_ value of 131 ± 21 μM for UDP-ManNAc and a V_max_ value of 0.159 ± 0.021 μmol/min/mg protein were determined for the reverse reaction (S12B Fig). The reversible reaction attained an equilibrium ratio of 9:1 in favor of UDP-GlcNAc, in line with reported epimerization ratios ranging from 12:1 to 9:1 for homologous enzymes [52, 64,] including the orthologous MnaA 2-epimerase required for *B*. *subtilis* WTA biosynthesis [51]. Testing tunicamycin in the *in vitro* system revealed a dose-dependent inhibition of MnaA (Fig 8), verifying that the 2-epimerase indeed represents a secondary target within the WTA biosynthesis pathway.

## Discussion

Here, we describe genetic, biochemical, and X-ray crystal structure studies revealing the functional role of MnaA and Cap5P, encoding 2-epimerases which interconvert UDP-GlcNAc and UDP-ManNAc and provide the requisite substrate for the two first enzymes involved in Staphylococcal WTA biosynthesis, TarO and TarA, respectively. Whereas most of the enzymes involved in WTA polymer synthesis have been extensively characterized, the role of 2-epimerases in this process has remained largely enigmatic amongst medically relevant *Staphylococci*. Presumably, this is due to the genetic redundancy between MnaA and Cap5P in *S*. *aureus* and the limited studies of WTA biogenesis performed in *S*. *epidermidis*, where its identification is uniquely amenable by genetic means. To identify the functional contribution of MnaA in Staphylococcal WTA biosynthesis, L638, a recently discovered WTA inhibitor with potent *S*. *epidermidis* activity was used as a chemical probe to screen for novel bypass suppressor mutations able to reverse the drug’s bacteriostatic effect [33]. Extensive genetic and chemical biology evidence predict that in addition to target-based drug resistant mutations, additional bypass mutations may arise and reflect gene inactivation mutations in early non-essential steps in WTA biosynthesis [33, 34]. Accordingly, bypass mutations in *tarO* and *tarA* as well as *mnaA* were uncovered by L638^R^ suppressor analysis and WGS of resistor isolates. Indeed, an extensive characterization of *mnaA* and *cap5P* mutant phenotypes in both MRSA and MRSE described here reveal that WTA 2-epimerases serve as a new and highly unconventional class of antibiotic drug targets.

Unlike traditional antibiotic drug targets, MnaA and other early stage WTA enzymes are not essential for cell growth or viability. In fact, genetic inactivation of MnaA 2-epimerase activity resulted in only a minimal effect on Staphylococcal growth rate. However, *mnaA* mutant phenotypes faithfully recapitulate those of *tarO* and *tarA* mutants and reveal multiple therapeutic contexts in which a cognate inhibitor to MnaA could provide broad efficacy against methicillin-resistant *Staphylococci*. Firstly, we demonstrate these 2-epimerases are essential for WTA synthesis in both MRSA and MRSE and depletion of WTA dramatically restores β-lactam susceptibility to these drug resistant pathogens both *in vitro* as well as in relevant mouse infection models. Therefore, as we and others have proposed [8, 16, 17, 33, 45, 65–67], inhibitors to such β-lactam potentiation targets could serve as novel adjuvants to partner with existing β-lactams to restore bactericidal therapeutic activity against β-lactam resistant *Staphylococci*. We also provide extensive evidence that abolishing WTA biosynthesis renders methicillin-resistant *Staphylococci* unable to effectively form robust biofilms. Accordingly, inhibitors of early stage WTA biosynthetic enzymes, including MnaA, may also serve as prophylactic agents to prevent *Staphylococcal* biofilm formation. As inhibitors of any of these targets are not expected to display antibacterial activity, such prophylactic agents are also predicted to be highly selective and spare the gut microbiota from antibiotic-mediated alterations. Finally, as *ΔtarO* strains exhibit dramatically attenuated virulence phenotypes across diverse animal infection models tested [35, 55, 68], WTA 2-epimerase inhibitors may also provide prophylactic or therapeutic utility as novel anti-virulence agents.

Surprisingly, S. *epidermidis* encodes only a single epimerase whereas *S*. *aureus* encodes two related enzymes amongst all published genomes we have examined. This likely reflects that *S*. *aureus* expresses a second epimerase involved in capsular biosynthesis, which is not produced by *S*. *epidermidis*. Indeed, *S*. *aureus* Cap5P epimerizes UDP-GlcNAc and UDP-ManNAc and participates in CP5 synthesis [52, 69]. Therefore, whereas *S*. *epidermidis* MnaA appears solely responsible for WTA synthesis, *S*. *aureus* requires a second enzyme to fulfill the biosynthetic needs of two disparate cell wall polymers. Interestingly, *B*. *anthracis* also requires two highly related 2-epimerases to fulfill biogenesis of the S-Layer, a cell wall elaboration analogous to WTA in many ways, and one member (GneZ) is essential for vegetative growth [70].

Biochemical analysis of MnaA revealed that the 2-epimerase interconverts UDP-GlcNAc and UDP-ManNAc, demonstrating reversible epimerase activity. At equilibrium the conversion of UDP-GlcNAc to UDP-ManNAc attained ~10%, thereby limiting the available amount of UDP-ManNAc for WTA biosynthesis. This agrees well with the required prioritization of UDP-GlcNAc for the essential processes of peptidoglycan biosynthesis within the cell. MnaA may thus resemble a checkpoint that contributes to control the flux of UDP-GlcNAc and channel the shared soluble cell wall precursor into the different synthesis pathways.

As TarO, MnaA and Cap5P all bind a common substrate, UDP-GlcNAc, we examined whether the 2-epimerases potentially serve as additional WTA targets of tunicamycin. Tunicamycin is a natural product-derived antibiotic that is structurally related to UDP-GlcNAc and can bind in the active sites of TarO [47] and MraY [16, 62]. We demonstrate that tunicamycin also binds to purified MnaA and Cap5P by STD NMR and inhibits the MnaA catalyzed interconversion of UDP-GlcNAc and UDP-ManNAc in a dose-dependent fashion *in vitro*. These findings may explain the tremendous potency of tunicamycin as a WTA inhibitor versus a PG inhibitor. Whereas tunicamycin displays a relatively low activity against *S*. *aureus* (MIC = 32 μg ml^-1^), it is a highly potent WTA inhibitor (IC_50_ = 50 ng ml^-1^) [16]. We speculate this preferential inhibitory activity of WTA over PG biosynthesis may in part reflect that tunicamycin inhibits both TarO and MnaA/Cap5P-mediated steps in WTA biosynthesis and that even partial inhibition of each enzyme in the pathway may be synergistic in a whole cell context, analogous to the synergistic mechanism of combining trimethoprim (TMP) and sulfonamide (SULF) antibiotics [5]. Unlike the chemical synergy achieved by TMP and SULF, however, tunicamycin may achieve this effect due to its polypharmacological effects as a single agent. Notably, a single agent antibiotic with multiple targets is predicted to possess a very low propensity for drug resistance, which is true for tunicamycin. Therefore, a non-toxic analog of tunicamycin, alternative active site inhibitor, or allosteric inhibitor of TarO and MnaA/Cap5P may all benefit by displaying potent WTA inhibitory activity as well as an extremely low frequency of resistance. Finally, due to the limited homology between MnaA and the closest human BLASTP homolog, GNE2 (S2B Fig), it is unlikely that host 2-epimerases would be affected.

Based on the crystal structure of the *B*. *anthracis* UDP-GlcNAc 2-epimerase, an *in silico* screen was recently performed to identify a UDP-GlcNAc 2-epimerase inhibitor named epimerox [71, 72]. Although several chemotypes of epimerox are reported to recapitulate terminal phenotypes of the *B*. *anthracis* epimerase conditional mutant, no direct genetic, biochemical, biophysical, or structural data are provided to independently reinforce this conclusion. Surprisingly, epimerox displays potent *S*. *aureus* activity (MIC = 8 μg ml^-1^) [71] and *S*. *epidermidis* activity (MIC = 2 μM) [72] despite our conclusion that 2-epimerase activity is dispensable for growth in MRSA COL, MRSE CLB26329, and the routinely studied methicillin-sensitive *S*. *aureus* strain, RN4220 (S13 Fig). Such a paradox between the bioactivity of epimerox and non-essentiality of its reported drug target in multiple different strain backgrounds suggest that additional mechanism of action studies seem warranted, including whether epimerox effectively inhibits WTA synthesis, synergizes in combination with β-lactams, and/or prevents biofilm formation or Staphylococcal virulence. Determining the *S*. *aureus* MnaA crystal structure as well as key residues essential for enzyme function offers important new resources to assist MnaA inhibitor discovery.

## Methods

### Strains, media, chemicals, growth conditions

MRSA COL is a hospital-acquired penicillinase-negative strain extensively used in *Staphylococcus aureus* methicillin resistance and virulence studies [73, 74] and from which its genome has been fully sequenced and annotated [75]. MRSE strain (MB6255) is a previously described methicillin-resistant *S*. *epidermidis* clinical isolate (CLB26329; [76]) isolated from a New York ICU in 2004. All strains were grown in trypticase soy broth (TSB) or cation-adjusted Mueller Hinton broth (CAMHB) (Difco, BD, Franklin Lakes, New Jersey, USA) at 37°C, 250rpm unless otherwise indicated. All compounds were prepared in DMSO. All strains are described in Table C in S1 Text. All subcloning methods are described in S1 Text.

### Isolation and genetic confirmation of LOF mutants

Approximately 1 x 10^9^ cells of strains MRSE CLB26329, MRSA COL or *Δcap5P* grown to stationary phase overnight were spread on CAMHA (Difco) containing 16 μg ml^-1^ L638 for MRSE (4-fold MIC) and 8 μg ml^-1^ L638 (4-fold MIC) for MRSA and *Δcap5P*. Plates were incubated for 48–96 hours for MRSE and 48–72 hours for MRSA and *Δcap5P*. L638 resistance was confirmed in a second round of growth on 16 μg ml^-1^ L638, and colonies were counter screened against 8 μg ml^-1^ imipenem to differentiate mutations in TarG versus early and intermediate steps in WTA biosynthesis. Genomic DNA was prepared from imipenem sensitive mutants (DNEasy Blood & Tissue Kit, Qiagen, Venlo, Netherlands) and Sanger sequencing for *mnaA* was performed using *mnaA*-locus specific primers 1731, 1732, 1733 and 1734 for MRSE and primers 1525 and 1526 for MRSA (Table B in S1 Text). Sequence analysis was performed using Sequencher 5.0 software. MnaA LOF mutations were independently confirmed by Illumina-based whole genome sequencing (>100× genome coverage) (BGI Hong Kong). No additional non-synonymous mutations were found in MRSE. Only one MRSA COL LOF mutant carried an additional non-synonymous mutation (Fig 2).

### Susceptibility testing

MICs were determined by the broth microdilution method in accordance with the recommendations of the Clinical and Laboratory Standards Institute in 96 well plates and assayed visually. MRSA strains were tested in CAMHB (Difco). MRSE strains were tested in Luria Bertani broth (Difco).

### WTA extraction and WTA-PAGE

Previously published [26]. Very briefly, stationary phase cells were used for extractions. Cells were washed and boiled for one hour, and pellets harvested for further processing. WTA was hydrolyzed and run on polyacrylamide gel electrophoresis.

### Mouse deep thigh model

Performed as previously published [53]. Briefly, immune-suppressed CD-1 mice (5 per group) were challenged intramuscularly in the right thigh with 1x10^6^ CFUs of MRSA for imipenem efficacy or with indicated 10-fold dilutions for virulence studies. Mice were challenged with 2x10^6^ CFUs of MRSE. For efficacy studies, mice were treated with indicated amounts of imipenem (IPM). Thighs were harvested at 24hrs, homogenized and plated to determine CFU per thigh.

### Ethics statement

All animal procedures were performed in accordance with the highest standards for the humane handling, care and treatment of research animals and were approved by the Merck Institutional Animal Care and Use Committee. The care and use of research animals at Merck meet or exceed all applicable regulations of the Animal Welfare Act as put forth by the United States Department of Agriculture. The protocol number is 2018-300643-Jan. It was approved in January of 2015 and will expire in January of 2018.

### Biofilm assays

For total biofilm formation assays, wild-type MRSA COL or MRSE CLB26329 and their derived loss of function mutants or MRSA COL *ltaS* hypomorph were grown in TSB (Difco) with or without sub-MIC concentrations of drugs overnight at 37°C, 250rpm. Cultures were normalized to OD600 = 1.5 and diluted 1/50 in TSB + 0.2% glucose with or without indicated sub-MIC concentrations of drug. 200μl of culture were seeded in triplicate wells in duplicate 96-well plates pretreated overnight with bovine plasma (Lampire, Pipersville, Pennsylvania, USA). Plates were incubated wrapped in parafilm at 37°C for 24 hours. One plate was shaken to resuspend biofilm and pellicles in liquid and OD_600_ taken to quantify total growth per well. The duplicate plate was processed for biofilm analysis. Supernatant was aspirated and wells washed gently three times with H_2_O. Biofilms were then fixed with Bouin’s fixative (Electron Microscopy Sciences, Hatfield, Texas, USA) for 15 minutes, supernatant removed and biofilms stained with 0.1% safranin (Ricca Chemical Company, Arlington, Texas) solution for 15 minutes. Plates were washed under running tap water to remove excess stain. Stained biofilms were dissolved in glacial acetic acid and OD_564_ measurements taken to quantify biofilm formation. Readings were normalized to corresponding total growth readings from the duplicate plate.

For biofilm killing assays, biofilms were grown for 24 hours as above in the absence of compounds before addition of compounds at indicated drug concentrations. Biofilms were incubated another 24 hours, washed, fixed and stained with Syto 10 (Life Technologies, Carlsbad, California, USA) for total cell staining and DEAD Red (Life Technologies) for membrane-damaged, dead cell staining. Plates were excited at 492nm and emissions read at 505 and 615nm, respectively.

For fluorescence microscopy, biofilms were grown for 24 hours as above in Cellcoat black *μ*Clear 96 well plates (Greiner Bio-one, Monroe, North Carolina, USA) in the absence or presence of compounds, supernatant was aspirated and wells washed gently three times with H_2_O. Biofilms were then stained with 0.1 μM *Bac*Light Green bacterial stain (Life Technologies) in DPBS for 15 minutes, washed once and fixed with 4% formaldehyde for 30 minutes. Biofilms were examined at 60x magnification on a Nikon Eclipse T*i* using a FITC filter. Z-stacks were acquired using NIS Elements AR software (Nikon, Tokyo, Japan).

### Crystal structure and LOF modeling

Protein purification methods are described in Supplemental materials. The *S*. *aureus* COL MnaA protein sample concentrated at 31 mg ml^-1^ was screened for crystallization by free interface diffusion using Topaz nano-chips. The crystallization condition most readily transferable to a set-up by vapor diffusion contained 0.1M Na Cacodylate, pH 6.5, 0.1M Li_2_SO_4_, 30% PEG 400. However the reproducibility of the experiments was poor and the crystals when they grew had often poor diffraction not exceeding 4–6 Å. Structure determination using the best crystal showed that UDP is present bound to the protein in the crystal, although no exogenous UDP was ever added to the protein sample at any step during protein purification or crystallization. Crystallization reproducibility and crystal diffraction were subsequently significantly improved by adding Na_2_UDP from a 200 mM stock solution to the protein sample right before crystallization set-up to a final ligand concentration of 4 mM. The final optimized conditions were 0.1 M Tris Cl pH 8.0, 0.1M Na_2_SO_4_, 52% PEG 400 at a temperature of 22°C, adding 1.5 μl precipitant to 1.5 μl protein and let equilibrate by vapor diffusion against precipitant in a hanging drop set-up. For diffraction experiments the crystals were harvested from the crystallization drop and directly frozen in a bath of liquid nitrogen.

The crystals grow in space group P 2_1_ 2_1_ 2_1_, a = 55.5Å, b = 85.8Å, c = 168.8Å with two molecules per asymmetric unit and diffract up to 1.9Å. The data were collected at the Canadian Light Source 08ID-1 beam line on a Mar mosaic CCD300 CCD detector (Canadian Light Source, Saskatoon, Saskatchewan, Canada). The diffraction data were processed, reduced and merged using the autoPROC automated pipeline with calls to the XDS software for indexing and integration, and the package AIMLESS for scaling and merging. The structure was solved by molecular replacement with the MOLREP program using PDB entry 1F6D as a starting point. The model was first refined with autoBUSTER, then rebuilt with the sequence switched to the *S*. *aureus* sequence using the COOT graphical suite. The structure was compared with one rebuilt at that point with the Phenix automated AutoBuild procedure and some results obtained with the latter incorporated in the model. The UDP ligand was added in a difference “omitmap” (i.e. the ligand was always excluded from the model prior to generating this map). After several cycles of refinement using autoBUSTER and rebuilding with COOT the final models contains all residues except residues 38 to 43, 60 to 67, and from 376 to the C-terminal residue in one copy of the molecule in the asymmetric unit, and 60 to 67, and from 376 to the C-terminal end in the other. The model also contains one molecule of UDP per chain, 185 waters, and 3 sulfate anions. It refines to a final crystallographic R_work_ and R_free_ values of 20.4% and 22.7%, respectively, and presents good stereochemistry according to the program Molprobity. The model and structure factors have been deposited in the Protein Data Bank with code 5ENZ.

### NMR binding experiments

Tunicamycin binding was detected by saturation transfer difference (STD) NMR. 15 μM tunicamycin was added into 500 μl binding buffer [25 mM Tris(d_11_)-DCl (pD 8.0), 50 mM NaCl and 25 μM TSP (2,2,3,3-Tetradeutero-3(trimethylsilyl)propionic acid) in 99.98% D_2_O] which contained either 5 μM 2-epimerase (MnaA/Cap5P) or no protein as a negative control. The binding mixture was incubated for 2 hours at 25°C before NMR data collection. STD NMR spectra were collected at 298 K on a Bruker 600 MHz Avance spectrometer (Bruker, Billerica, Massachusetts, USA) equipped with a 5 mm TXI cryogenic probe. Selective saturation of the protein was applied by switching the on-and off-resonance saturation frequency after each scan. A train of Gaussian shape pulses with 50 ms pulse length (corresponding to an excitation width of 100 Hz) separated by a delay of 1ms was used, with the total length of the selective saturation set to 3s, and the on-and off-resonance saturation frequencies set to -120 Hz and 20,000 Hz, respectively. A total time of 50 minutes was required to collect a single STD NMR spectrum including sample changing. The STD NMR experiment was repeated on a solution of tunicamycin in the absence of protein to exclude any artifacts and make sure the observed STD NMR signals are due to tunicamycin binding to 2-epimerase.

### Assays for enzymatic activity of MnaA

MnaA-catalyzed interconversion of UDP-GlcNAc and UDP-ManNAc was carried out in a total volume of 50 μl containing either UDP-GlcNAc or UDP-ManNAc (0–3 mM as indicated) in 10 mM NaP_i_, 50 mM NaCl, pH 8.0. Reactions were initiated by the addition of 0.109 μg MnaA-His_6_ (forward reaction, FW) or 0.327 μg MnaA-His_6_ (reverse reaction, RV) and incubated for 10 min to 5 h at 30°C. All enzymatic reactions were quenched by heating (10 min, 100°C) and analyzed by capillary electrophoresis. Tunicamycin (Sigma Aldrich, Munich, Germany) was added at concentrations ranging from 0 to 200 μM. Reactions were stopped by heating after 120 min (10 min, 100°C).

## Supporting Information

S1 Text Supporting materials and methods. Detailed description of materials, methods, supporting references and supporting Tables A-C.
(DOCX)

S1 Fig MnaA loss of function mutants in MRSA and MRSE fail to produce WTA. WTA extraction and SDS PAGE analysis from MRSA COL mutants (left) and L638^R^ MRSE CLB26329 (right). Note, wild-type MRSA WTA polymers appear as a ladder of discretely sized bands whereas a more diffuse staining of MRSE WTA polymer is observed. WTA material was normalized to cell biomass prior to loading. The *tarO*_*Sa*_^Y95^* deletion mutant serves as a control for complete impairment of WTA polymer production. Deletion of *cap5P* did not noticeably affect WTA production.
(TIF)

S2 Fig Cap5P and MnaA share a high degree of homology, and only MnaA is present in *S*. *epidermidis*. (A) Alignment of MRSA COL MnaA and Cap5P and MRSE CLB26329 MnaA demonstrates high degrees of sequence identity and similarity. Black shading represents identical and grey is similar using default consensus settings with the BOXSHADE program (http://sourceforge.net/projects/boxshade/). (B) Alignment of MRSA COL MnaA with its closest human homolog, GNE2. GNE2 has 2 domains, the N-terminal part in its longest isoform shows 22% sequence identity. Alignment was done in Clustal Omega program [S12]. An asterisk indicates positions which have a single, fully conserved residue. A colon indicates conservation between groups of strongly similar properties—scoring > 0.5 in the Gonnet PAM 250 matrix. A period indicates conservation between groups of weakly similar properties—scoring = < 0.5 in the Gonnet PAM 250 matrix.
(TIF)

S3 Fig Generation of *cap5P* deletion mutant in MRSA COL. (A) Suicide plasmid pSAKO was modified for replication in *S*. *aureus* by cloning the temperature sensitive replicon *repF* from plasmid pAUL-A, into the Sac1 restriction site, yielding pSAKO^TS^. (B) To create the ziracin resistance cassette using 3-way PCR, the *emtA* gene was PCR amplified from plasmid pPAM19 with primers 1700+1701. Approximately 1 kB of *cap5P* upstream sequence was PCR amplified from MRSA COL with primers 1702+1704, appending an Aatll restriction site. Similarly, *cap5P* downstream sequence was amplified with primers 1703+1646, appending a Bglll site. (C) The 3 fragments of the *cap5P*::*emtA* cassette were stitched together using primers 1704+1646, restriction digested with Aatll and Bglll, then ligated and cloned into pSAKO^TS^, yielding the final *cap5P*::*emtA* knockout plasmid.
(TIF)

S4 Fig Representative growth curves of MRSA COL and MRSE CLB26328 epimerase mutants. Growth in 20 mL cultures was monitored by viable counts at 0, 2, 4, 6 and 24 hours. (A) Growth of COL mutants *Δcap5P* and *Δcap5P mnaA*_*Sa*_^*P12L*^ in Mueller Hinton broth compared to *ΔtarO* and isogenic parent control strain. (B) Growth of *Δcap5P mnaA*_*Sa*_^*P12L*^ in MH broth + Chloramphenicol (CAM) 20 μg mL^-1^ and 0.5% xylose complemented by pEPSA5 vector alone or with pEPSA5 carrying *mnaA*_*Sa*_ or *cap5P*. (C) Growth of MRSE CLB26329 mutants (indicated) in LB broth and mutant complements in LB broth supplemented with CAM 20 μg mL^-1^ and 0.5% xylose.
(TIF)

S5 Fig MRSA COL *mnaA*, *Δcap5P* and double mutants display morphological defects consistent with WTA depletion. Structured illumination microscopy images of cells incubated with (A) Van-FL, and (B) Nile Red to label the cell wall and membrane, respectively. COL Δ*tarO* mutants lacking WTA show increased cell size heterogeneity, abnormal septal placement and cell separation defects (arrows), similar to the defects that result from the deletion of both epimerases. COL Δ*cap5P* mutants show a wild-type morphology. Scale bars represent 1 μm.
(TIF)

S6 Fig MRSE *mnaA* mutants display morphological defects consistent with WTA depletion. Structured illumination microscopy images of cells incubated with (A) Van-FL, and (B) Nile Red to label the cell wall and membrane, respectively. Mutants lacking WTA show increased cell size heterogeneity and septum placement defects (arrows). Scale bars represent 1 μm.
(TIF)

S7 Fig Imipenem is bactericidal against MRSA and MRSE lacking WTA 2-epimerase function. All strains were grown in the presence of 4 μg ml^-1^ imipenem (IPM) and CFUs measured over 24 hours. (A) MRSA COL and Δ*cap5P* are unaffected by imipenem (4 μg ml^-1^), while the Δ*cap5P mnaA*_*Sa*_^*P12L*^ mutant displays a 3 log reduction in cell viability within 7 hr of imipenem treatment. Plasmid-based copies of wild-type *cap5P* (p*cap5P*), *mnaA*_*Sa*_ (p*mnaA*_*Sa*_), and *mnaA*_*Se*_ (p*mnaA*_*Se*_) restore resistance to imipenem. (B) Kill curves of MRSE CLB26329 performed as in (A) using *mnaA*_*Se*_^*ΔY151*^ and 4 μg ml^-1^ imipenem plasmid-based copies of wild-type *cap5P* (p*cap5P*), *mnaA*_*Sa*_ (p*mnaA*_*Sa*_), and *mnaA*_*Se*_ (p*mnaA*_*Se*_) fully restore resistance to imipenem.
(TIF)

S8 Fig WTA is required for biofilm formation in MRSA. MRSA COL biofilms were grown in triplicates for 24 hours in 96-well black clear bottom plates with or without indicated sub-MIC concentrations of WTA inhibitors. Isolation and characterization of MRSA COL *tarO*, *tarA*, *tarB*, *tarD*, and *tarI* LOF mutants were described previously [34] and assayed here to broaden conclusions concerning the requirement of WTA in biofilm formation. Δ*pbp3*, Δ*pbp4*, and Δ*pbp3*, Δ*pbp4* double mutants are included as negative controls for the biofilm assay. Tunicamycin and L638 treatments were performed at the indicated sub-MIC drug concentrations. Genetic complementation of described mutants was performed using plasmid-based copies of wild-type *cap5P* (p*cap5P*), *mnaA*_*Sa*_ (p*mnaA*_*Sa*_), and *mnaA*_*Se*_ (p*mnaA*_*Se*_) as indicated. Biofilms were stained with *Bac*Light Green fluorescent stain. Z-stacks were obtained at 60x magnification. Scale bar = 10 μm.
(TIF)

S9 Fig WTA is required for biofilm formation in MRSE. MRSE CLB26329 biofilms were grown in triplicates for 24 hours in 96-well black clear bottom plates with or without indicated sub-MIC concentrations of WTA inhibitors. Isolation and characterization of MRSE *tarO*, and *tarA* LOF mutants were described previously [34] and assayed here to broaden conclusions concerning the requirement of WTA in biofilm formation. Tunicamycin and L638 treatments were performed at the indicated sub-MIC drug concentrations. Genetic complementation of described mutants was performed using plasmid-based copies of wild-type *cap5P* (p*cap5P*), *mnaA*_*Sa*_ (p*mnaA*_*Sa*_), and *mnaA*_*Se*_ (p*mnaA*_*Se*_) as indicated. Biofilms were stained with *Bac*Light Green fluorescent stain. Z-stacks were obtained at 60x magnification. Scale bar = 10 μm.
(TIF)

S10 Fig Inhibition of WTA biosynthesis cannot kill preformed biofilms. To assess biofilm killing by WTA inhibitors alone or in combination with imipenem at its clinical breakpoint, MRSA COL (A) and MRSE CLB26329 (B) biofilms were grown for 24 hours in 96-well plates before addition of WTA inhibitors tunicamycin (tuni), L638, in the absence or presence of imipenem (IPM), and then incubated for an additional 24 hours. Note drug concentrations of each inhibitor used are shown in parentheses as μg/ml. Biofilms were stained with Syto 10 for total cell staining and DEAD Red for membrane-damaged cells. Y-axis measures dead/live cell ratio. Error bars are standard deviations from triplicate experiments. No effect of any compounds tested alone or in combination was observed.
(TIF)

S11 Fig Representative capillary electropherogram of the MnaA-catalyzed interconversion of UDP-GlcNAc and UDP-ManNAc. Peaks, λmax = 260 nm: **1** buffer; **2** internal standard (I.S.) adenosine; **3** UDP-ManNAc; **4** UDP-GlcNAc.
(TIF)

S12 Fig Enzyme kinetics of the MnaA-catalyzed interconversion of UDP-GlcNAc and UDP-ManNAc. (A) Michaelis-Menten plot for MnaA forward reaction, K_m_ for UDP-GlcNAc: 411 ± 57 μM, V_max_: 0.171 ± 0.037 μmol/min/mg protein; (B) Michaelis-Menten plot for MnaA reverse reaction, K_m_: 131 ± 21 μM, V_max_: 0.159 ± 0.021 μmol/min/mg protein.
(TIF)

S13 Fig Methicillin-sensitive *S*. *aureus* RN4220 requires MnaA and Cap5P for WTA biosynthesis and biofilm formation. (A) WTA extraction and SDS PAGE analysis from MSSA RN4220 Δ*mnaA* and Δ*mnaA* Δ*cap5P*. Note, wild-type MSSA RN4220 WTA polymers appear as a ladder of discretely sized. WTA material was normalized to cell biomass prior to loading. Deletion of *mnaA* alone does not affect WTA levels, whereas the double deletion of *mnaA* and *cap5P* does. (B) For total biofilm quantification, biofilms were grown in triplicates for 24 hours in 96-well plates. Biofilms were stained with safranin and dissolved in glacial acetic acid before OD_564_ was measured. Bars represent mean OD, error bars represent standard deviation. Note that deletion of *mnaA* alone does not affect biofilm, whereas the double deletion of *mnaA* and *cap5P* does (C) MIC values of penicillin G, tetracycline, and L638. Note that deletion of *mnaA* alone does not confer resistance to TarG inhibitor L638, whereas the double deletion of *mnaA* and *cap5P* does.
(TIF)
