# Supplementary material for: Chemical Genetic Analysis and Functional Characterization of Staphylococcal Wall Teichoic Acid 2-Epimerases Reveals Unconventional Antibiotic Drug Targets
Source: PLoS Pathog. 2016 May 4;12(5):e1005585. doi: 10.1371/journal.ppat.1005585 (PMC4856313; doi:10.1371/journal.ppat.1005585)
Supplement: S1 Text — Detailed description of materials, methods, supporting references and supporting Tables A-C. (DOCX) [file ppat.1005585.s001.docx]

**Long Title: Chemical Genetic Analysis and Functional Characterization of Staphylococcal Wall Teichoic Acid 2-Epimerases Reveals Unconventional Antibiotic Drug Targets**

**Short Title: WTA 2-Epimerases are dual β-lactam Potentiation and antibiofilm Drug Targets**

**Supplemental Information**

**Methods**

**Construction of *Δcap5P* and *ΔtarO*.**

Suicide plasmid pSAKO [28] was modified for replication in *S. aureus* by cloning the temperature sensitive replicon *repF* from plasmid pAUL-A, a derivative of pE194 [S1] into the Sac1 restriction site, yielding pSAKO^TS^. The replicon was PCR amplified with primers 1612+1613, appending Sac1 sites on both ends. To create the ziracin resistance cassette using 3-way PCR, the *emtA* [S2] gene was PCR amplified from plasmid (pPAM19) with primers 1700+1701. Approximately 1 kB of *cap5P* upstream sequence was PCR amplified from MRSA COL with primers 1702+1704, appending an Aatll restriction site. Similarly, *cap5P* downstream sequence was amplified with primers 1703+1646, appending a Bglll site. The 3 fragments of the *cap5P::emtA* cassette were stitched together using primers 1704+1646, restriction digested with Aatll and Bglll, then ligated and cloned into pSAKO^TS^. (S3 Fig) All *E. coli* transformations were performed using chemically competent DH10B cells (Invitrogen, Waltham, Massachusetts, USA) and following the manufacturer’s protocols. All PCR amplifications were performed using Takara’s ExTaq polymerase. To generate the *cap5P* knockout in *S. aureus*, appx 0.2 µg of pSAKO^TS^ / *cap5::emtA* plasmid was electroporated into *S. aureus* strain RN4220 using the methods of Lee [S3]. Following electroporation, cells were plated on MHA containing 10 µg ml^-1^ of ziracin plus 20 µg ml^-1^ of Kanamycin and incubated for 48 hours at the permissive temperature 30°C. To select for single crossover integration at the *cap5P* locus, two resistant isolates were grown in MHB containing 10 µg ml^-1^ ziracin for 2 hours at 30°C, then shifted to 42°C for 6 hours and plated on MHA plus 10 µg ml^-1^ ziracin and incubated overnight at 42°C. To select for the double crossover *cap5P* knockout and suicidal loss of plasmid, individual colonies were streaked onto to MHA + ziracin 10 µg ml^-1^ + 10% sucrose and incubated overnight at 37°C. These Kan^S^ isolates were demonstrated by PCR to contain the desired *Δcap5P::emtA* deletion. The *cap5P* KO was subsequently transduced into *S. aureus* COL using phage 80, selecting on MHA + ziracin 10 µg ml^-1^ and incubating at 37°C for 48 hours. The *ΔtarO* construction was performed similarly in pSAKO using restriction sites Sph1 and Nco1 with primers 1551-1556. Primers are listed in Table B in S1 Text. All primers were obtained from Sigma-Aldrich Oligos (Sigma-Aldrich, Saint Louis, Missouri, USA).

**Construction of *S. aureus Δpbp3Δpbp4*** **mutant.**

To construct a *pbp3* null mutant, we amplified 1kb DNA fragments from *S. aureus* COL spa- genomic DNA, corresponding to the upstream (primers pPBP3-KO-P1 and pPBP3-KO-P2) and downstream (primers pPBP3-KO-P3 and pPBP3-KO-P4) regions of the *pbp3* gene. The resulting PCR products were joined by overlap PCR using primers PBP3-KO-P1 and PBP3-KO-P4. The overlap PCR product was digested with EcoRI and BamHI and cloned into the thermosensitive plasmid pMAD [S4], producing the plasmid p*Δpbp3*. The plasmid was sequenced and introduced into RN4220 by electroporation [S5]. Following electroporation the plasmid was transduced using phage 80α to COL spa- [S6] using a previously described method [S7]. Insertion and excision of pΔ*pbp3* into the chromosome was performed as previously described [S4], resulting in the strain *Δpbp3.* For in-frame deletion of *pbp4*, encoding PBP4, 1kb from the upstream and downstream regions of the gene were amplified by PCR using primer pairs pPBP4-KO-1/pPBP4-KO-2 and pPBP4-KO-3/pPBP4-KO-4, respectively. These fragments were joined by overlap PCR using primers pPBP4-KO-1 and pPBP4-KO-4, digested with NcoI and BamHI and cloned into pMAD [S4], resulting in plasmid p*Δpbp4*. The plasmid was introduced into strains COL spa- and COL spa- *Δpbp3*, integration and excision of the plasmid to delete *pbp4* was performed as described [S4], resulting in strains *Δpbp4* and *Δpbp3Δpbp4*. All gene deletions were confirmed by PCR using primer pairs pPBP3-KO-P5/P6 and pPBP4-KO-P5/P6 and sequencing.

**Super-resolution Structured illumination microscopy (SIM).**

Overnight cultures of parental and mutant strains were diluted 1/500 and incubated at 37°C. At mid-exponential phase (OD_600nm_ 0.5), 1 ml from each culture was taken and incubated with the membrane dye Nile Red (10 µg ml^-1^, Invitrogen) and the cell wall dye Van-FL (Molecular Probes) mixed in a 1/1 (v/v) proportion with non-fluorescent vancomycin (Sigma), to a final concentration of 4 µg ml^-1^, at room temperature for 5 minutes with shaking. Cells were harvested by centrifugation, re-suspended in PBS and 1 µl was placed on a thin layer of 1.2 % agarose in PBS. 2D-SIM images were obtained using a Zeiss Elyra PS.1 microscope with a sCMOS Pro camera, using Zen software (Zeiss). Twenty-five images were taken (5 phases x 5 rotations) in two channels (561nm; 34 μm grating period, 488nm; 28 μm grating period) and reconstructions for each channel were performed using Zen software and a theoretical PSF (point spread function).

**Homology searches.**

We compared all currently available NCBI public genomes to infer presence of *mnaA* and *cap5P* in *S. aureus* (4308 genomes) and *S. epidermidis* (105 genomes) using BLASTP 2.2.17 [S8].

**Construction of 2-epimerase complemented LOF mutants.**

Complementation plasmids were constructed by amplifying the native genes from either *S. aureus* COL or *S. epidermidis* ATCC 14990 genomic DNA using Phusion polymerase (New England Biolabs, Ipswich, Massachusetss, USA) and inserting them downstream of the xylose inducible promoter of the *S. aureus* expression vector pEPSA5 [S9] using restriction endonucleases SacI and SalI. Expression of genes cloned into pEPSA5 can be induced with 0.5% xylose or can be repressed by addition of 0.5% glucose. To introduce the plasmids into *S. epidermidis* CLB 26329 and to complement the strains *Δcap5P mnaA_Sa_^P12L^*, *Δcap5P mnaA_Sa_^Y194*^*, *mnaA_Se_^ΔY151^* and *mnaA_Se_^G171D^*, various amounts (0.25-3 µg) of complementation plasmids purified from *E. coli* DC10B [S10] were electroporated into these strains. Briefly, 50 µl competent cells, prepared as described previously [S10] were mixed with plasmid DNA, incubated at room temperature for 30 min, transferred into a 1-mm gap electroporation cuvette and pulsed at 2.1 kilovolts. Immediately after electroporation, 1 ml TSB (Difco) was added followed by a 90 min incubation period at 37°C. Subsequently, different dilutions of cells were plated on LB agar containing 34 µg ml^-1^ of chloramphenicol and incubated for 24-48 hours at 37°C. Plasmids were introduced into *S. aureus* COL and *S. aureus* COL Δ*cap5P* by phage transduction as described previously [S11] using bacteriophage 85 lysate from *S. aureus* RN4220. To inactivate the host restriction system, cells were heat shocked for 2 min at 52°C prior to infection.

**Kill Curves.**

Strains were grown overnight from frozen stock in CAMHB (Difco) at 37˚C, 250rpm. 20ml of CAMHB in vented cap 125ml flasks were inoculated with 1:10000 *S. aureus* cultures or 1:7500 *S. epidermidis* cultures and grown at 37˚C, 70rpm for one hour. Imipenem was added to final concentration of 4µg ml^-1^. CFUs were plated onto trypticase soy agar supplemented with 5% sheep’s blood at concentrations ranging from 0 to 10^-7^ by taking an aliquot from each of the samples and diluting in sterile 0.9% saline at 0, 2, 4, 8 & 24 hrs.

**Growth Curves.**

*Staphylococcus aureus* isolates were cultured overnight to stationary phase in cation-adjusted Mueller Hinton Broth (MHB); *Staphylococcus epidermidis* isolates were cultured overnight in Miller’s LB broth. After spectrophotometer normalization, 20 mL of pre-warmed MHB was inoculated with *S. aureus* to an approximate cell density of 5.5 x 10 ^5^ cells ml^-1^; *S. epidermidis* was similarly inoculated into pre-warmed LB broth. All cultures were incubated at 37°C, shaking at 200 rpm. Cultures with isolates complemented with either the pEPSA5 vector alone, *MnaA* or *Cap5P* were supplemented with Chloramphenicol 20 µg ml^-1^ and 0.5% xylose. At time points of 0, 2, 4, 6, and 24 hours, 0.5 mL was sampled from each flask and diluted appropriately (10^-2^ to 10^-6^) in 0.9% saline to obtain countable colony forming units (CFU). Diluted cells were plated on TSA II 5% sheep blood (BBL) in a spiral gradient using a Neutec Eddy Jet. Plates were imaged and CFU ml^-1^ were determined using a Neutec Flash and Go colony counter. The LOG10 CFU ml^-1^ were plotted as a function of time using Excel.

**Generation of *mnaA* and *cap5P* sequential knockouts in MSSA RN4220.**

Temperature sensitive suicide plasmid pSAKO^TS^ was used to carry an unmarked deletion in the *mnaA* coding region. Amino acid residues 101 through 200 were targeted for deletion using two-way PCR. Approximately 0.7 kB of *mnaA* upstream and 0.3 kB of N-term coding sequence was PCR amplified from RN4220 with primers 1774+1775, appending a Bglll restriction site. Similarly, sequence corresponding to AA residues 201 to the end of the *mnaA* coding region and 570 nucleotides beyond was amplified with primers 1776+1777, appending a Sall restriction site. To create overlap with the first PCR fragment, 30 nucleotides, corresponding to residues 91 through 100, were added to the 5’ end of primer 1776. The two overlapping fragments were stitched together with ExTaq polymerase using primers 1774+1777, restriction digested with Bglll and Sall, then cloned into pSAKO^TS^. This deletion plasmid was used to electrotransform [S3] RN4220 to Kan resistance, selected at the permissive 30°C temperature. To select for single crossover integration at the *mnaA* locus, two resistant isolates were grown in MHB containing 20 µg ml^-1^ Kan for 2 hours at 30°C, then shifted to 42°C for 6 hours and plated on MHA plus 20 µg ml^-1^ Kan and incubated overnight at 42°C. To select for the double crossover *mnaA* knockout and suicidal loss of plasmid, individual colonies were streaked onto MHA + 10% sucrose and incubated overnight at 37°C. Eight single colonies were selected for genome preparation and PCR KO confirmation; two of the eight isolates were confirmed to have the correct *ΔmnaA* deletion. To generate the *ΔmnaA/cap5P::emtA* double deletion, the pSAKO^TS^/*cap5::emtA* plasmid was used to transform single deletion RN4220/*ΔmnaA,* then resolved to the *cap5P* KO as described previously in this manuscript.

**Protein Expression and Purification.**

The *S. aureus* COL *mnaA* and *cap5P* genes with a C-terminal GG linker+HIS8 tag sequence was cloned into pDEST14 vector. The recombinant *S. aureus* COL MnaA protein was expressed in *E. coli* strain BL21(DE3) Star in Terrific Broth (Difco) supplemented with 50 μg ml^-1^ ampicillin for 18 hours at 16°C with 1 mM IPTG.  The recombinant *S. aureus* Cap5P protein was expressed in *E. coli* strain BL21(DE3) Star in Super Broth supplemented with 100 μg ml^-1^ carbenicillin for 4hours at 30°C with 0.2 mM IPTG. The cells were harvested by centrifugation for 15 minutes at 6000 × g.  Pellets were then resuspended in 50 mM Tris, 0.5 M NaCl, 10% glycerol, 1 mM DTT, and 1 mg ml^-1^ protease inhibitor cocktail III at pH 8.0 and then lysed with a microfluidizer.  The cell lysate was clarified by centrifugation at 100,000 × g for 1 hour at 4°C.  The supernatant was filtered and loaded onto a Ni2+-IMAC column (Qiagen) equilibrated with 50 mM Tris, 0.5 M NaCl, 10% glycerol, and 1 mM DTT at pH 8.0.  The protein was eluted using an imidazole gradient (0 to 0.25 M) containing 50 mM Tris, 0.5 M NaCl, 10% glycerol, and 1 mM DTT at pH 8.0.  Fractions containing MnaA protein were pooled and concentrated for further purification by size exclusion chromatography using a Superdex 200 26/60 column (GE Healthcare, Princeton, NJ, USA).  The final storage buffer for MnaA protein was 50 mM Hepes and 0.25 M NaCl, 5% glycerol at pH 7.5.  Fractions with > 95% pure peptides (as monitored by sodium dodecyl sulfate polyacrylamide gel electrophoresis (SDS-PAGE)) were collected and concentrated to 10-20 mg ml^-1^ using a centrifugal concentrator.  The identity of the protein was confirmed by electrospray ion trap mass spectrometry (ESI-Ion-Trap-MS) using a LTQ-XL mass spectrometer (ThermoScientific, Rockford, IL, USA) and the Xcalibur software platform (ThermoScientific, Rockford, IL, USA).

**X-ray structures comparison and figure generation.**

All superpositions were performed using the LSQMAN program [S14]. Atoms which were further away than 3.5Å (the default) after superposition were not included in the positional R.M.S.D calculation. All figures showing a projection of a three-dimensional structure were created using the PyMol software [S15].

**Capillary electrophoresis.**

All experiments were carried out using a P/ACE MDQ capillary electrophoresis system (Beckman Instruments, Fullerton, CA, USA) equipped with a diode array detector (DAD). The capillary temperature was kept constant at 25 °C. The electrophoretic separations were carried out by using a fused-silica capillary of 40 cm total length (30 cm effective length) × 75.5 µm (id) × 363.7 µm (od) obtained from Optronis GmbH. The method is based on published CE methods [S16-18]. The following conditions were applied: λ_max_ = 260 nm, voltage = 20 kV, running buffer 40 mM borax buffer, pH 9.1 (adjusted by boric acid), hydrodynamic injection (0.5 psi, 5 sec). The capillary was washed with 0.2 M NaOH (1 min) and running buffer (2 min) before each injection. Data collection and corrected peak area analysis were performed by the 32 Karat software obtained from Beckman coulter (Fullerton, CA, USA). Further data analysis was carried out by Graph Pad Prism 4 (Graph Pad Software, Inc. California). 50 µl of a 20 µM adenosine stock solution in 4 mM borax, pH 10, were added to the analytical samples as an internal standard (I.S.). The linearity of the quantitative determination, the limit of detection (LOD) and the limit of quantification (LOQ) of the enzymatic products UDP-ManNAc and UDP-GlcNAc were determined by processing five-point calibration curves (triplicate samples) in the presence of adenosine as I.S. A strictly linear correlation between analyte concentration and peak-area ratio of analyte to I.S. was observed for the determined concentrations ranging from 5 µM to 100 µM of UDP-ManNAc.

**Determination of kinetic parameters for MnaA.**

To determine the linear range of the enzymatic reactions, interconversion of UDP-GlcNAc and UDP-ManNAc was monitored over time (9 time points, 10 min to 5 h) at 30°C with a substrate concentration of 500 µM (UDP-GlcNAc), or 100 µM (UDP-ManNAc), respectively. Negative controls were performed in the presence of heat-inactivated enzyme (10 min, 100°C). For determination of kinetic parameters (K_m_ and V_max_), 8 (FW) and 10 (RV) different substrate concentrations were chosen. Each analysis was repeated three times in independent experiments with duplicate measurements.

**Supplemental References**

S1. Williams PH, Ketley J, Salmond G **(**1998) Bacterial Pathogenesis, Volume 27 of Methods in Microbiology, Academic Press pp: 427-430

S2. Mann PA, Xiong L, Mankin AS, Chau AS, Mendrick CA, Najarian DJ, et al. (2001) EmtA, a rRNA methyltransferase conferring high-level evernimicin resistance. Mol Microbiol 41(6): 1349-56.

S3. Lee JC. 1995. Electrotransformation of Staphylococci. Methods Mol Biol 47**:** 209-16.

S4. Arnaud M, Chastanet A, Debarbouille M (2004) New vector for efficient allelic replacement in naturally nontransformable, low-GC-content, gram-positive bacteria. Appl Environ Microbiol 70: 6887-6891.

S5. Veiga H, Pinho MG (2009) Inactivation of the SauI type I restriction-modification system is not sufficient to generate *Staphylococcus aureus* strains capable of efficiently accepting foreign DNA. Appl Environ Microbiol 75: 3034-3038. doi: 10.1128/AEM.01862-08.

S6. Reed P, Veiga H, Jorge AM, Terrak M, Pinho MG (2011) Monofunctional transglycosylases are not essential for *Staphylococcus aureus* cell wall synthesis. J Bacteriol 193: 2549-2556. doi: 10.1128/JB.01474-10.

S7. Oshida T, Tomasz A (1992) Isolation and characterization of a Tn551-autolysis mutant of *Staphylococcus aureus*. J Bacteriol 174: 4952-4959.

S8. Altschul SF, Madden TL, Schäffer AA, Zhang J, Zhang Z, Miller W, et al. (1997) Gapped BLAST and PSI-BLAST: a new generation of protein database search programs**.** Nucleic Acid Res 25(17):3389-402.

S9. Forsyth RA, Haselbeck RJ, Ohlsen KL, Yamamoto RT, Xu H, Trawick JD, et al. (2002) A genome-wide strategy for the identification of essential genes in *Staphylococcus aureus*. Mol Microbiol 43(6):1387-400.

S10. Monk IR, Shah IM, Xu M, Tan MW, Foster TJ (2012) Transforming the untransformable: application of direct transformation to manipulate genetically *Staphylococcus aureus* and *Staphylococcus epidermidis.* MBio 3(2) pii: e00277-11. doi: 10.1128/mBio.00277-11.

S11. Berger-Bächi B (1983) [Increase in transduction efficiency of Tn551 mediated by the methicillin resistance marker.](http://www.ncbi.nlm.nih.gov/pubmed/6300040) J Bacteriol 154(1):533-5.

S12. Sievers F, Higgins DG (2014) Clustal Omega. Curr Protoc Bioinformatics. 48:3.13.1-3.13.16. doi: 10.1002/0471250953.bi0313s48.

S13. Kreiswirth BN, Löfdahl S, Betley MJ, O'Reilly M, Schlievert PM, Bergdoll MS, et al. (1983) The toxic shock syndrome exotoxin structural gene is not detectably transmitted by a prophage. Nature 305(5936):709-12.

S14. Kleywegt GJ, Jones, TA. (1994) CCP4/ESF-EACBM Newsletter on Protein Crystallography. A super position 31:9-14.

S15. Delano W (2002) The PyMol molecular graphics system version 1.7 Schrödinger, LLC.

S16. K. O’Riordan, J.C. Lee, Staphylococcus aureus capsular polysaccharides., Clin. Microbiol. Rev. 17 (2004) 218–34.

S17. I.C. Schoenhofen, D.J. McNally, E. Vinogradov, D. Whitfield, N.M. Young, S. Dick, et al., Functional characterization of dehydratase/aminotransferase pairs from Helicobacter and Campylobacter: enzymes distinguishing the pseudaminic acid and bacillosamine biosynthetic pathways., J. Biol. Chem. 281 (2006) 723–32.

S18. L. Zhang, M.M. Muthana, H. Yu, J.B. McArthur, J. Qu, X. Chen, Characterizing non-hydrolyzing Neisseria meningitidis serogroup A UDP-N-acetylglucosamine (UDP-GlcNAc) 2-epimerase using UDP-N-acetylmannosamine (UDP-ManNAc) and derivatives, Carbohydr. Res. 419 (2016) 18–28.

**Table A. MnaA loss of function mutants in MRSA and MRSE specifically exhibit restored β-lactam susceptibility.**

|  | **MIC (µg ml^-1^)** | | | | | |
| --- | --- | --- | --- | --- | --- | --- |
| **Strain** | **IPM** | **Naf** | **Dic** | **L638** | **Vanco** | **Tuni** |
| MRSA COL | 32 | > 64 | > 64 | 2 | 2 | > 64 |
| Δ*cap5P* | 32 | > 64 | > 64 | 2 | 2 | > 64 |
| Δ*cap5P mnaA_Sa_^P12L^* | 1 | 8 | 4 | 16 | 2 | > 64 |
| Δ*cap5P mnaA_Sa_^E287G^* | 1 | 8 | 2 | 16 | 2 | > 64 |
| Δ*cap5P mnaA_Sa_^Y194G^* | 1 | 8 | 2 | 16 | 2 | > 64 |
| Δ*cap5P mnaA_Sa_^247FS^* | 1 | 8 | 2 | 16 | 2 | > 64 |
| Δ*cap5P mnaA_Sa_^T97M^* | 1 | 8 | 2 | 16 | 2 | > 64 |
| Δ*cap5P mnaA_Sa_^Q286*^* | 0.5 | 4 | 2 | 16 | 2 | > 64 |
| Δ*cap5P mnaA_Sa_^R136S^* | 0.5 | 4 | 2 | 16 | 2 | > 64 |
| Δ*cap5P mnaA_Sa_^T203K^* | 0.5 | 8 | 2 | 16 | 2 | > 64 |
| Δ*cap5P mnaA_Sa_^E245*^* | 0.5 | 8 | 2 | 16 | 2 | > 64 |
| Δ*cap5P mnaA_Sa_^214FS^* | 0.5 | 8 | 4 | 16 | 2 | > 64 |
| Δ*cap5P mnaA_Sa_^D281Y^* | 0.5 | 8 | 4 | 16 | 2 | > 64 |
| MRSE CLB26329 | 64 | > 64 | > 64 | 4 | 4 | 16 |
| *mnaA_Se_^R300I^* | 0.5 | 0.25 | 0.125 | 16 | nd | nd |
| *mnaA_Se_^76::12bp^* | 4 | 0.125 | 0.125 | 16 | nd | nd |
| *mnaA_Se_^P129T^* | 0.25 | 0.25 | 0.125 | 16 | nd | nd |
| *mnaA_Se_^ΔY151^* | 0.25 | 0.25 | 0.125 | 16 | 2 | 16 |
| *mnaA_Se_^P129T^* | 0.5 | 0.25 | 0.125 | 16 | nd | nd |
| *mnaA_Se_^P131L^* | 0.125 | 0.125 | 0.125 | 16 | nd | nd |
| *mnaA_Se_^G283R^* | ≤ 0.063 | 0.25 | 0.25 | 16 | nd | nd |
| *mnaA_Se_^D281E^* | ≤ 0.063 | 0.125 | 0.125 | 4 | nd | nd |
| *mnaA_Se_^G171D^* | 0.25 | 0.125 | 0.125 | 16 | 2 | 16 |

Additional *mnaA* and *cap5P* mutations isolated by L638^R^ selection in MRSA and MRSE. MIC values of β-lactams imipenem (IPM), nafcillin (Naf), and dicloxacillin (Dic). L638 is included to quantify drug resistance of bypass mutations, vancomycin and tunicamycin are included as non-β-lactam controls.

**Table B. Primers and plasmids used in this study.**

| **Primer sequence** | **Primer #** | **target / description** |
| --- | --- | --- |
| GTCGTCGAGCTCGCATCACACGCAAAAAGGA | 1612 | *repF* TS origin of replication with Sac1 site, forward |
| GTCGTCGAGCTCGCTGGTGCGAAAAAAGAGTGT | 1613 | *repF* TS origin of replication with Sac1 site, reverse |
| CAGCAGCAGACGTCCATTGCCGTTCCGACGCCGAA | 1704 | *cap5P* upstream flanking region with Aatll site, forward |
| AAACATTTCCTCATGTTTTAAAAGCTGATCAACATTATCCTCTCTGAAGTTCAAACACA | 1702 | *cap5P* upstream region, reverse |
| GATCAGCTTTTAAAACATGAGGA | 1700 | *emtA* region, resistance cassette,forward |
| CTACTTCCTTCTAAGTCCATCTTGT | 1701 | *emtA* region resistance cassette, reverse |
| TGTTTGAAAAACAAGATGGACTTAGAAGGAAGTAGGATGAATTCGTACCTTTACGTCACA | 1703 | *cap5P* downstream flanking region, forward |
| GTGGTGAGATCTCAGCTCACAGAAGTCTCA | 1646 | *cap5P* downstream flanking region with Bglll site, reverse |
| CAGCAGCAGCGATATCGATTAATAATAATGCGATACT | 1551 | *tarO* upstream flanking region with Sph1 site, forward |
| TTTCCTCATGTTTTAAAAGCTGATCTATTATAAATTAATTCGTTCAATC | 1552 | *tarO* upstream flanking region, reverse |
| TTGATTGAACGAATTAATTTATAATAGATCAGCTTTTAAAACATGAGGAAA | 1553 | *emtA* region, resistance cassette,forward |
| CAGCTATGCTTTCATTCCCTATTCCTCCTACTTCCTTCTAAGTCCATCTTGT | 1556 | *emtA* region resistance cassette, reverse |
| ACAAGATGGACTTAGAAGGAAGTAGGAGGAATAGGGAATGAAAGCATAGCTG | 1555 | *tarO* downstream flanking region, forward |
| CAGCAGCAGCCATGGCCTGCGACAGATAACTTGTAGA | 1554 | *tarO* downstream flanking region with Nco1 site, reverse |
| GCTGCACTTGATGAAAAGCT | 1525 | *S. aureus mnaA* sequencing, forward |
| CCGTTCCACTCATACAAACT | 1526 | *S. aureus mnaA* sequencing, reverse |
| CACCAGGTTTAGGTGATGCA | 1731 | *S. epidermidis mnaA* sequencing, forward |
| CGTTCCACTCATACAATCT | 1732 | *S. epidermidis mnaA* sequencing, reverse |
| GCTATCACTGGTAATACAGCT | 1733 | *S. epidermidis mnaA* internal sequencing, forward |
| CTTGTATTCCACCAGAGTCA | 1734 | *S. epidermidis mnaA* internal sequencing, reverse |
| GCGCGGAGCTCATGAAAAAAATTATGGTTATTTTCG | Am1 | *cap5P* amplification with SacI site, forward |
| GCGCGGTCGACTTATTTGTGACGTAAAGGTACG | Am2 | *cap5P* amplification with SalI site, reverse |
| GCGCGGAGCTCATGAAAAAGATTATGACCATATTTG | Am3 | *mnaA_SA_* amplification with SacI site, forward |
| GCGCGGTCGACTCAGAAATCGCTTGGTTTTTCA | Am4 | *mnaA_SA_* amplification with SalI site, reverse |
| GCGCGGAGCTCTTGATGAAAAAAGTTATGACCATAT | Am5 | *mnaA_SE_* amplification with SacI site, forward |
| GCGCGGTCGACTTATAAATCTTTTGTAAATTCAAAATG | Am6 | *mnaA_SE_* amplification with SalI site, reverse |
| TATTTGCTTTCAGGAAAATTTTTC | Am7 | pEPSA5 sequencing primer, forward |
| TCTGTTTTATCAGACCG | Am8 | pEPSA5 sequencing primer, reverse |
| GTGGTGGTGAGATCTGCGTTGCGAAATTAACAGCTGA | 1774 | upstream *mnaA* region with Bglll site, forward |
| TGTCGTCATCGTATCACCATGT | 1775 | upstream *mnaA* region, reverse |
| ACCAGACATGGTACTTGTACATGGTGATACGATGACGACACTTATGACAGCACACCGACGA | 1776 | downstream mnaA region, forward |
| GTGGTGGTGGTCGACACTCACGAGCGGGGATTTGTGATCCA | 1777 | downstream mnaA region with Sall site, reverse |
| **Description of Plasmid** | **Name** | **Reference** |
| *S. aureus* plasmid carrying inducible resistances | pAUL-A | Williams et al (1998) Academic Press |
| *S. aureus* suicide vector utilized containing *sacB* | pSAKO | D’Elia et al (2006a) J Bacteriol |
| pSAKO containing a *S. aureus* *tarO* deletion cassette, *emtA* | pSAKO / *tarO::emtA* | This work |
| pSAKO containing temperature-sensitive *repF*for *S. aureus* replication | pSAKO^TS^ | This work |
| pSAKO^TS^ containing a *S.aureus cap5p* deletion cassette, *emtA* | pSAKO^TS^ / *cap5::emtA* | This work |
| *E. coli-S. aureus* shuttle vector containing a xylose inducible promoter; Apr Cmr | pEPSA5 | Forsyth et al (2002) Mol Microbiol |
| pEPSA5 containing *cap5P* amplified from *S. aureus* COL | pEPSA5-*capP_SA_* | This work |
| pEPSA5 containing *mnaA* amplified from *S. aureus* COL | pEPSA5-*mnaA_SA_* | This work |
| pEPSA5 containing *mnaA* amplified from *S. epidermidis* ATCC 14490 | pEPSA5-*mnaA_SE_* | This work |

**Table C. Bacterial strains used in this study.**

| **Strain Description** | **Name** | **Reference** |
| --- | --- | --- |
| Δ*dcm* in *E. coli* DH10B background; Dam methylation only | DC10B | Monk et al (2012) *MBio* |
| Restriction-deficient derivative of *S. aureu*s NCTC8325-4 | RN4220 | Kreiswirth et al (1983) *Nature* |
| methicillin-resistant clinical isolate of *S. epidermidis* | CLB26329 | Huber et al (2009) *Chem Biol* |
| hospital-acquired penicillinase-negative *S. aureus*strain | MRSA COL | Gill et al (2005) *J Bacteriol* |
| MRSA COL with mutation Y95* in *tarO,* isolated on TarG inhibitor | *tarO_Sa_^Y95*^* | Wang et al (2013) *Chem Biol* |
| MRSA COL with a frameshift at position 148 of *tarA,* isolated onTarG inhibitor | *tarA_Sa_^148FS^* | Wang et al (2013) *Chem Biol* |
| MRSA COL with mutation T295* in *tarB,* isolated onTarG inhibitor | *tarB_Sa_^T295*^* | Wang et al (2013) *Chem Biol* |
| MRSA COL with a frameshift at position 73 of *tarD,* isolated onTarG inhibitor | *tarD_Sa_^73FS^* | Wang et al (2013) *Chem Biol* |
| MRSA COL with mutation D234G in *tarI',* isolated onTarG inhibitor | *tarI'_Sa_^D234G^* | Wang et al (2013) *Chem Biol* |
| MRSA COL, *cap5P* has been replaced with *emtA* | Δ*cap5P* | This work |
| MRSA COL, *tarO* has been replaced with *emtA* | Δ*tarO* | This work |
| MRSA COL transposon mutant with lowered LtaS expression | *ltaS* | Meredith et al (2012) *Mob Genet Elements* |
| CLB26329 with mutation R300I in MnaA, isolated on TarG inhibitor | *mnaA_Se_^R300I^* | This work |
| CLB26329 with mutation P129T in MnaA, isolated on TarG inhibitor | *mnaA_Se_^P129T^* | This work |
| CLB26329 with deletion of residue 151 of MnaA, isolated on TarG inhibitor | *mnaA_Se_*^Δ^*^151^* | This work |
| CLB26329 with a 4 amino acid insertion at position 76 in MnaA, isolated on TarG inhibitor | *mnaA_Se_^76::12bp^* | This work |
| CLB26329 with mutation P131L in MnaA, isolated on TarG inhibitor | *mnaA_Se_^P131L^* | This work |
| CLB26329 with mutation G283R in MnaA, isolated on TarG inhibitor, isolated on TarG inhibitor | *mnaA_Se_^G283R^* | This work |
| CLB26329 with mutation D281E in MnaA, isolated on TarG inhibitor | *mnaA_Se_^D281E^* | This work |
| CLB26329 with mutation G171D in MnaA, isolated on TarG inhibitor | *mnaA_Se_^G171D^* | This work |
| CLB26329 with mutation D32Y in TarO, isolated on TarG inhibitor | *tarO_Se_^D32Y^* | This work |
| Δ*cap5P* with mutation P12L in MnaA, isolated on TarG inhibitor | Δ*cap5P mnaA_Sa_^P12L^* | This work |
| Δ*cap5P* with mutation E287G in MnaA, isolated on TarG inhibitor | Δ*cap5P mnaA_Sa_^E287G^* | This work |
| Δ*cap5P* with mutation Y194* in MnaA, isolated on TarG inhibitor | *Δcap5P mnaA_Sa_^Y194*^* | This work |
| Δ*cap5P* with a frameshit at position 247 of MnaA and mutation P654L in PTS/IIABC, isolated on TarG inhibitor | Δ*cap5P mnaA_Sa_^247FS^* | This work |
| Δ*cap5P* with mutation T97M in MnaA, isolated on TarG inhibitor | Δ*cap5P mnaA_Sa_^T97M^* | This work |
| Δ*cap5P* with mutation Q286* in MnaA, isolated on TarG inhibitor | Δ*cap5P mnaA_Sa_^Q286*^* | This work |
| Δ*cap5P* with mutation T203K in MnaA, isolated on TarG inhibitor | Δ*cap5P mnaA_Sa_^R136S^* | This work |
| Δ*cap5P* with mutation R136S in MnaA, isolated on TarG inhibitor | Δ*cap5P mnaA_Sa_^T203K^* | This work |
| Δ*cap5P* with mutation E245* in MnaA, isolated on TarG inhibitor | Δ*cap5P mnaA_Sa_^E245*^* | This work |
| Δ*cap5P* with a frameshit at position 214 of MnaA, isolated on TarG inhibitor | Δ*cap5P mnaA_Sa_^214FS^* | This work |
| Δ*cap5P* with mutation D281Y in MnaA, isolated on TarG inhibitor | Δ*cap5P mnaA_Sa_^D281Y^* | This work |
| Δ*cap5P* carrying pEPSA5 | Δ*cap5P /vector* | This work |
| Δ*cap5P* carrying pEPSA5-*mnaA_SA_* | Δ*cap5P /mnaA_Sa_* | This work |
| Δ*cap5P* carrying pEPSA5-*capP_SA_* | Δ*cap5P /cap5P_Sa_* | This work |
| Δ*cap5P* carrying pEPSA5-*mnaA_SE_* | Δ*cap5P /mnaA_Se_* | This work |
| Δ*cap5P mnaA_Sa_^P12L^* carrying pEPSA5 | Δ*cap5P mnaA_Sa_^P12L^/vector* | This work |
| Δ*cap5P mnaA_Sa_^P12L^* carrying pEPSA5-*mnaA_SA_* | Δ*cap5P mnaA_Sa_^P12L^/*p*mnaA_Sa_* | This work |
| Δ*cap5P mnaA_Sa_^P12L^* carrying pEPSA5-*cap5P_SA_* | Δ*cap5P mnaA_Sa_^P12L^/*p*cap5P_Sa_* | This work |
| Δ*cap5P mnaA_Sa_^P12L^* carrying pEPSA5-*mnaA_SE_* | Δ*cap5P mnaA_Sa_^P12L^/*p*mnaA_Se_* | This work |
| Δ*cap5P mnaA_Sa_^Y194*^* carrying pEPSA5 | *Δcap5P mnaA_Sa_^Y194*^/vector* | This work |
| Δ*cap5P mnaA_Sa_^Y194*^* carrying pEPSA5-*mnaA_SA_* | *Δcap5P mnaA_Sa_^Y194*^/*p*mnaA_Sa_* | This work |
| Δ*cap5P mnaA_Sa_^Y194*^* carrying pEPSA5-*cap5P_SA_* | *Δcap5P mnaA_Sa_^Y194*^/*p*cap5P_Sa_* | This work |
| Δ*cap5P mnaA_Sa_^Y194*^* carrying pEPSA5-*mnaA_SE_* | *Δcap5P mnaA_Sa_^Y194*^/*p*mnaA_Se_* | This work |
| *mnaA_Se_^ΔY151^* carrying pEPSA5 | *mnaA_Se_ ^ΔY151^/vector* | This work |
| *mnaA_Se_^ΔY151^* carrying pEPSA5-*mnaA_SA_* | *mnaA_Se_ ^ΔY151^/*p*mnaA_Sa_* | This work |
| *mnaA_Se_^ΔY151^* carrying pEPSA5-*cap5P_SA_* | *mnaA_Se_ ^ΔY151^/*p*cap5P* | This work |
| *mnaA_Se_^ΔY151^* carrying pEPSA5-*mnaA_SE_* | *mnaA_Se_ ^ΔY151^/*p*mnaA_Se_* | This work |
| *mnaA_Se_^G171D^* carrying pEPSA5 | *mnaA_Se_^G171D^/vector* | This work |
| *mnaA_Se_^G171D^* carrying pEPSA5-*mnaA_SA_* | *mnaA_Se_^G171D^/*p*mnaA_Sa_* | This work |
| *mnaA_Se_^G171D^* carrying pEPSA5-*cap5P_SA_* | *mnaA_Se_^G171D^/*p*cap5P* | This work |
| *mnaA_Se_^G171D^* carrying pEPSA5-*mnaA_SE_* | *mnaA_Se_^G171D^/*p*mnaA_Se_* | This work |
| MRSA COL with *pbp3* deletion | Δ*pbp3* | This work |
| MRSA COL with *pbp4* deletion | Δ*pbp4* | This work |
| MRSA COL with *pbp3* and *pbp4* deletions | Δ*pbp3 Δpbp4* | This work |
| RN4220 with unmarked *mnaA* deletion | RN4220 Δ*mnaA* | This work |
| RN4220 Δ*mnaA* in which *cap5P* has been replaced with *emtA* | RN4220 Δ*mnaA*Δ*cap5P* | This work |
| MRSA COL with mutation G84* in *tarO*, isolated onTarG inhibitor | *tarO_Se_^G84*^* | Wang et al (2013) Chem Biol |
| MRSA COL with mutation G129R of *tarA*, isolated onTarG inhibitor | *tarA_Se_^G129R^* | Wang et al (2013) Chem Biol |
