# Supplementary figures and images for: Chemical Genetic Analysis and Functional Characterization of Staphylococcal Wall Teichoic Acid 2-Epimerases Reveals Unconventional Antibiotic Drug Targets
Source: PLoS Pathog. 2016 May 4;12(5):e1005585. doi: 10.1371/journal.ppat.1005585 (PMC4856313; doi:10.1371/journal.ppat.1005585)

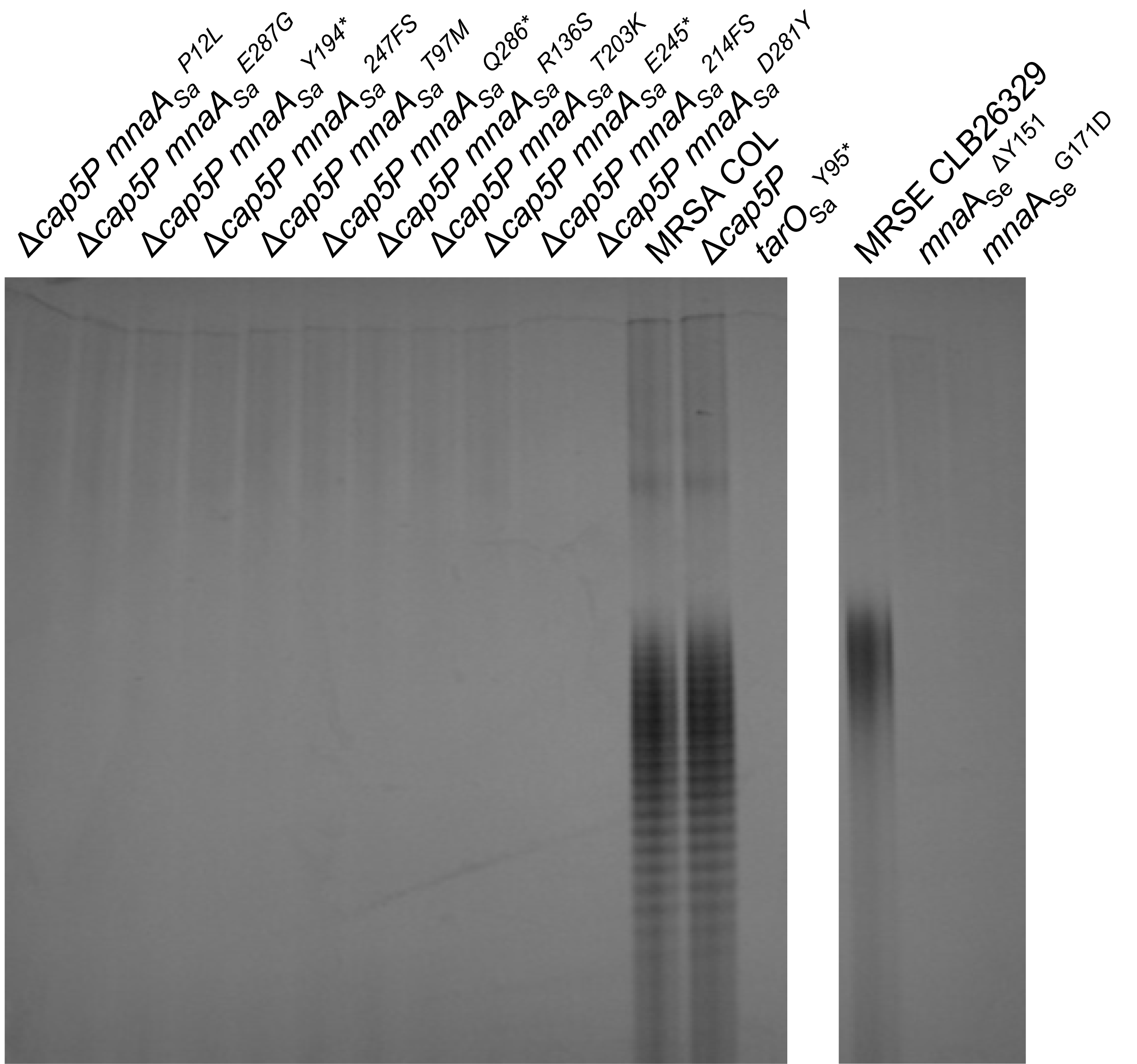

Supplement: S1 Fig — WTA extraction and SDS PAGE analysis from MRSA COL mutants (left) and L638R MRSE CLB26329 (right). Note, wild-type MRSA WTA polymers appear as a ladder of discretely sized bands whereas a more diffuse staining of MRSE WTA polymer is observed. WTA material was normalized to cell biomass prior to loading. The tarO Sa Y95* deletion mutant serves as a control for complete impairment of WTA polymer production. Deletion of cap5P did not noticeably affect WTA production. (TIF) [file ppat.1005585.s002.tif]

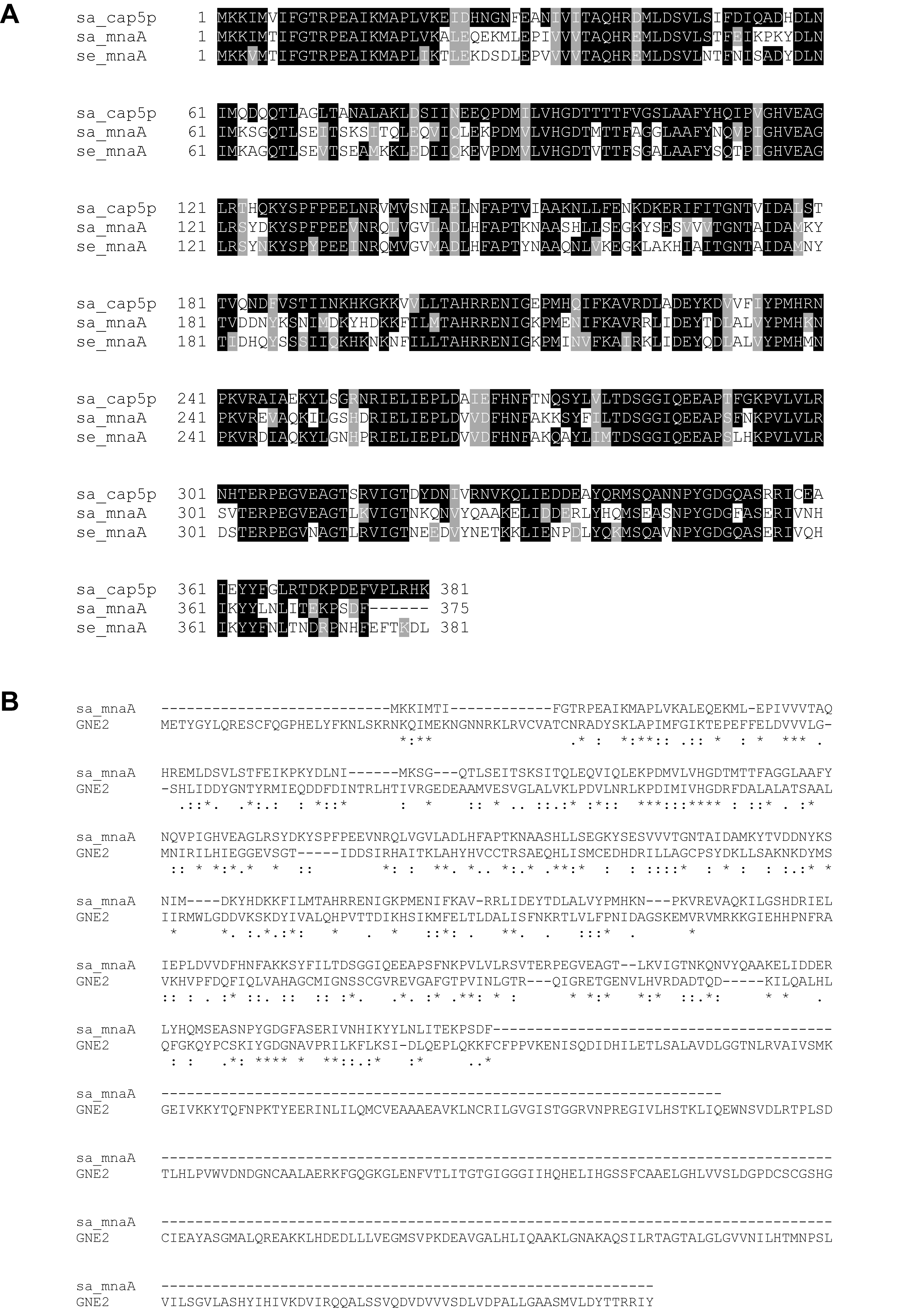

Supplement: S2 Fig — (A) Alignment of MRSA COL MnaA and Cap5P and MRSE CLB26329 MnaA demonstrates high degrees of sequence identity and similarity. Black shading represents identical and grey is similar using default consensus settings with the BOXSHADE program (http://sourceforge.net/projects/boxshade/). (B) Alignment of MRSA COL MnaA with its closest human homolog, GNE2. GNE2 has 2 domains, the N-terminal part in its longest isoform shows 22% sequence identity. Alignment was done in Clustal Omega program [S12]. An asterisk indicates positions which have a single, fully conserved residue. A colon indicates conservation between groups of strongly similar properties—scoring > 0.5 in the Gonnet PAM 250 matrix. A period indicates conservation between groups of weakly similar properties—scoring = < 0.5 in the Gonnet PAM 250 matrix. (TIF) [file ppat.1005585.s003.tif]

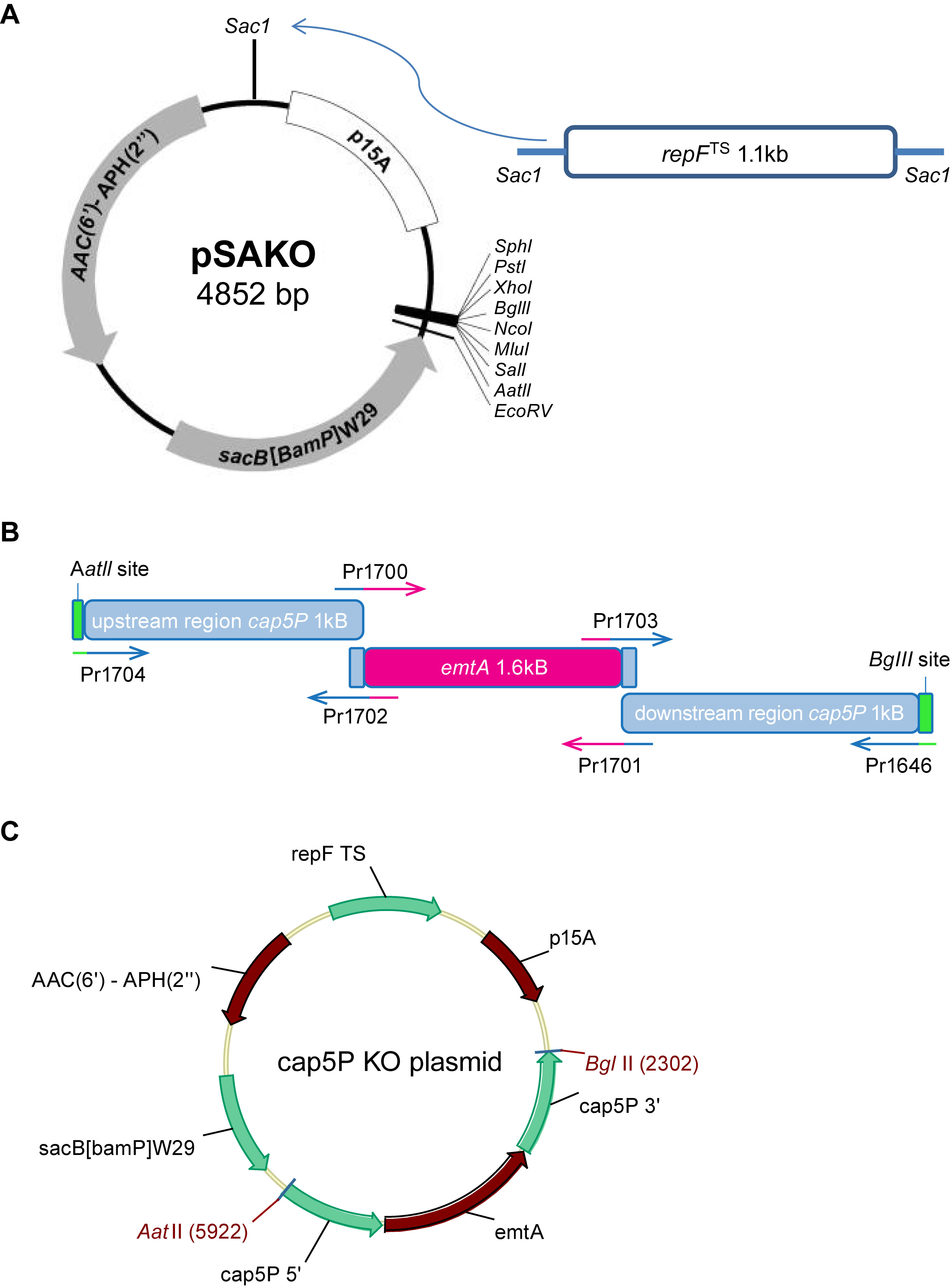

Supplement: S3 Fig — (A) Suicide plasmid pSAKO was modified for replication in S. aureus by cloning the temperature sensitive replicon repF from plasmid pAUL-A, into the Sac1 restriction site, yielding pSAKOTS. (B) To create the ziracin resistance cassette using 3-way PCR, the emtA gene was PCR amplified from plasmid pPAM19 with primers 1700+1701. Approximately 1 kB of cap5P upstream sequence was PCR amplified from MRSA COL with primers 1702+1704, appending an Aatll restriction site. Similarly, cap5P downstream sequence was amplified with primers 1703+1646, appending a Bglll site. (C) The 3 fragments of the cap5P::emtA cassette were stitched together using primers 1704+1646, restriction digested with Aatll and Bglll, then ligated and cloned into pSAKOTS, yielding the final cap5P::emtA knockout plasmid. (TIF) [file ppat.1005585.s004.tif]

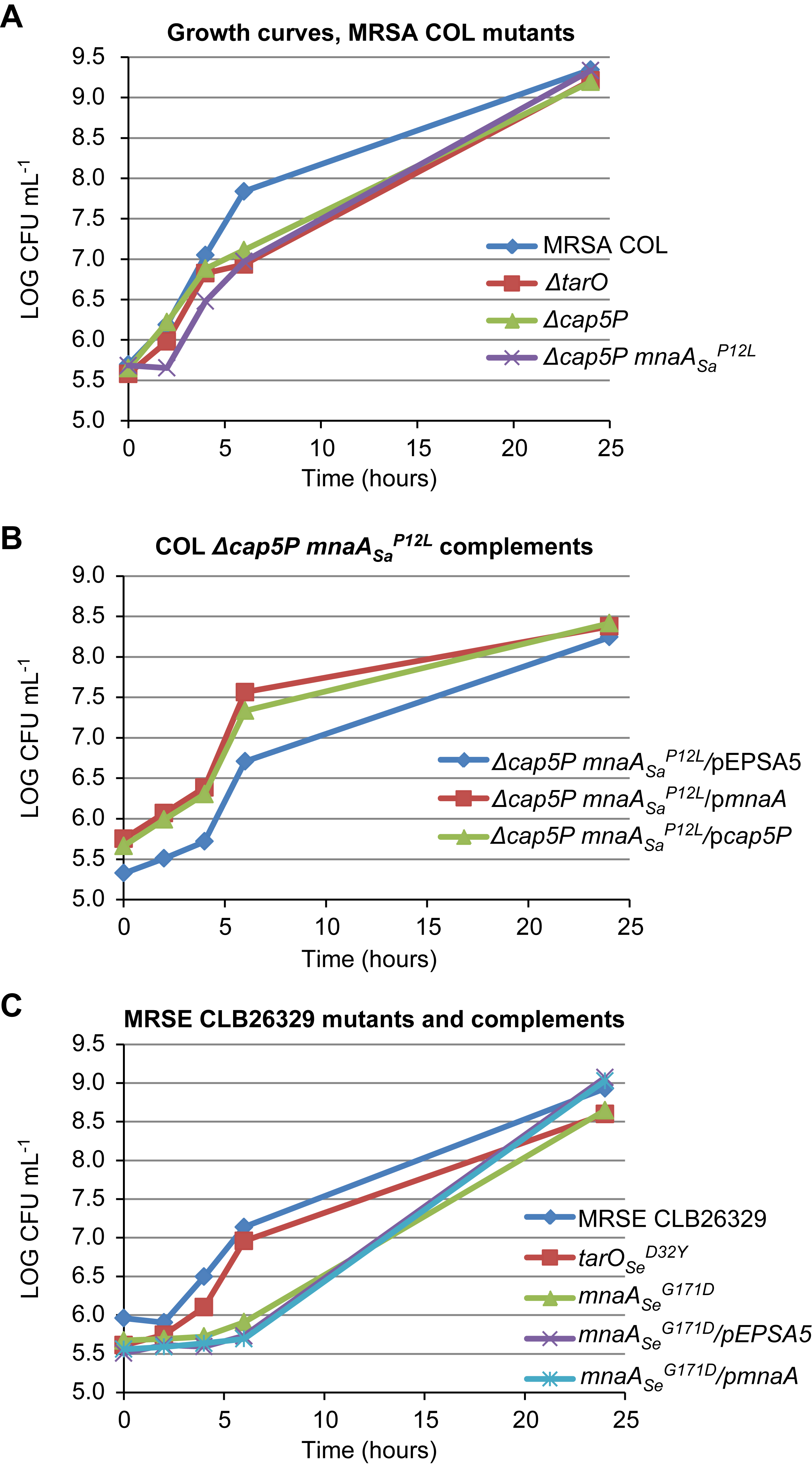

Supplement: S4 Fig — Growth in 20 mL cultures was monitored by viable counts at 0, 2, 4, 6 and 24 hours. (A) Growth of COL mutants Δcap5P and Δcap5P mnaA Sa P12L in Mueller Hinton broth compared to ΔtarO and isogenic parent control strain. (B) Growth of Δcap5P mnaA Sa P12L in MH broth + Chloramphenicol (CAM) 20 μg mL-1 and 0.5% xylose complemented by pEPSA5 vector alone or with pEPSA5 carrying mnaA Sa or cap5P. (C) Growth of MRSE CLB26329 mutants (indicated) in LB broth and mutant complements in LB broth supplemented with CAM 20 μg mL-1 and 0.5% xylose. (TIF) [file ppat.1005585.s005.tif]

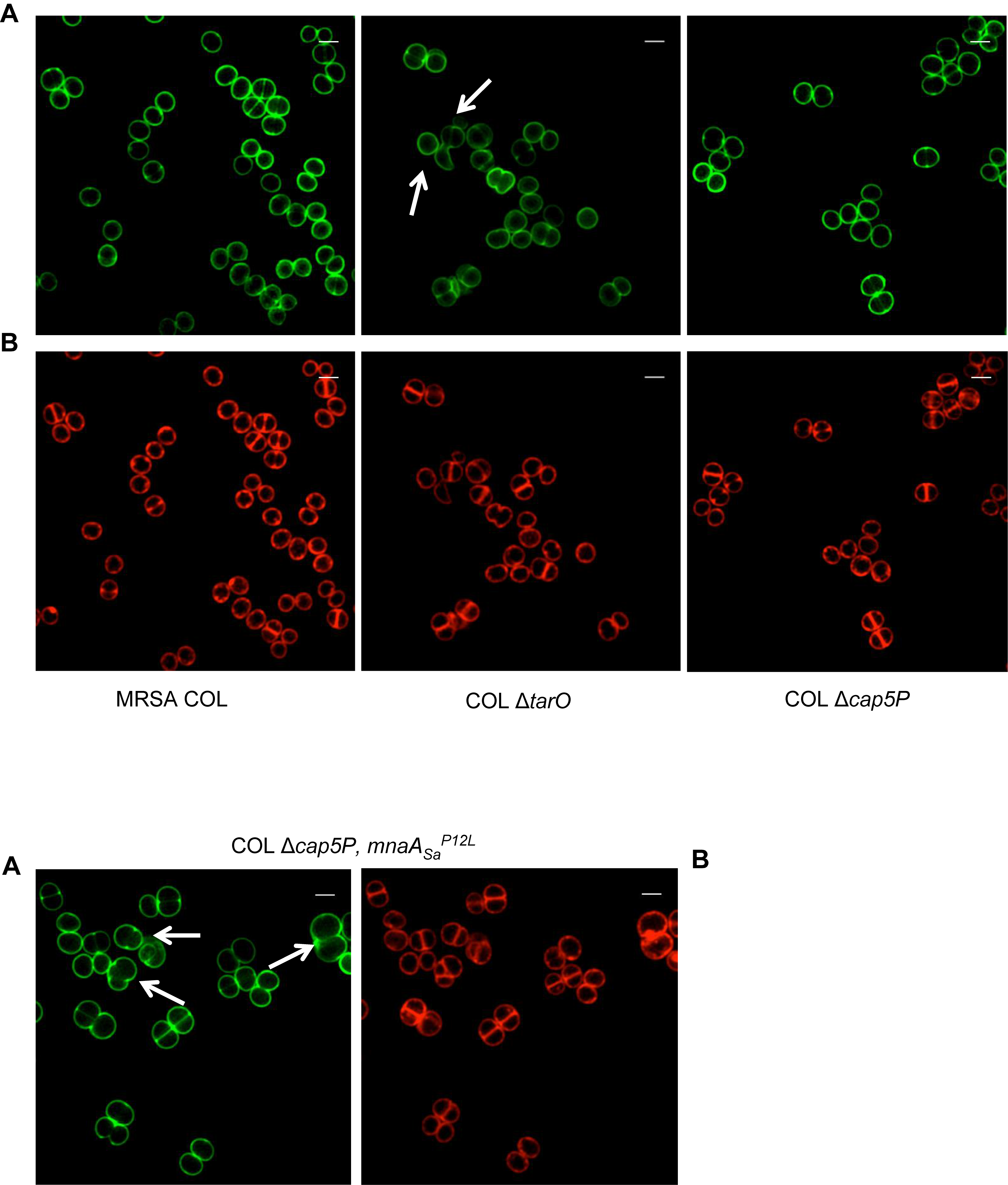

Supplement: S5 Fig — Structured illumination microscopy images of cells incubated with (A) Van-FL, and (B) Nile Red to label the cell wall and membrane, respectively. COL ΔtarO mutants lacking WTA show increased cell size heterogeneity, abnormal septal placement and cell separation defects (arrows), similar to the defects that result from the deletion of both epimerases. COL Δcap5P mutants show a wild-type morphology. Scale bars represent 1 μm. (TIF) [file ppat.1005585.s006.tif]

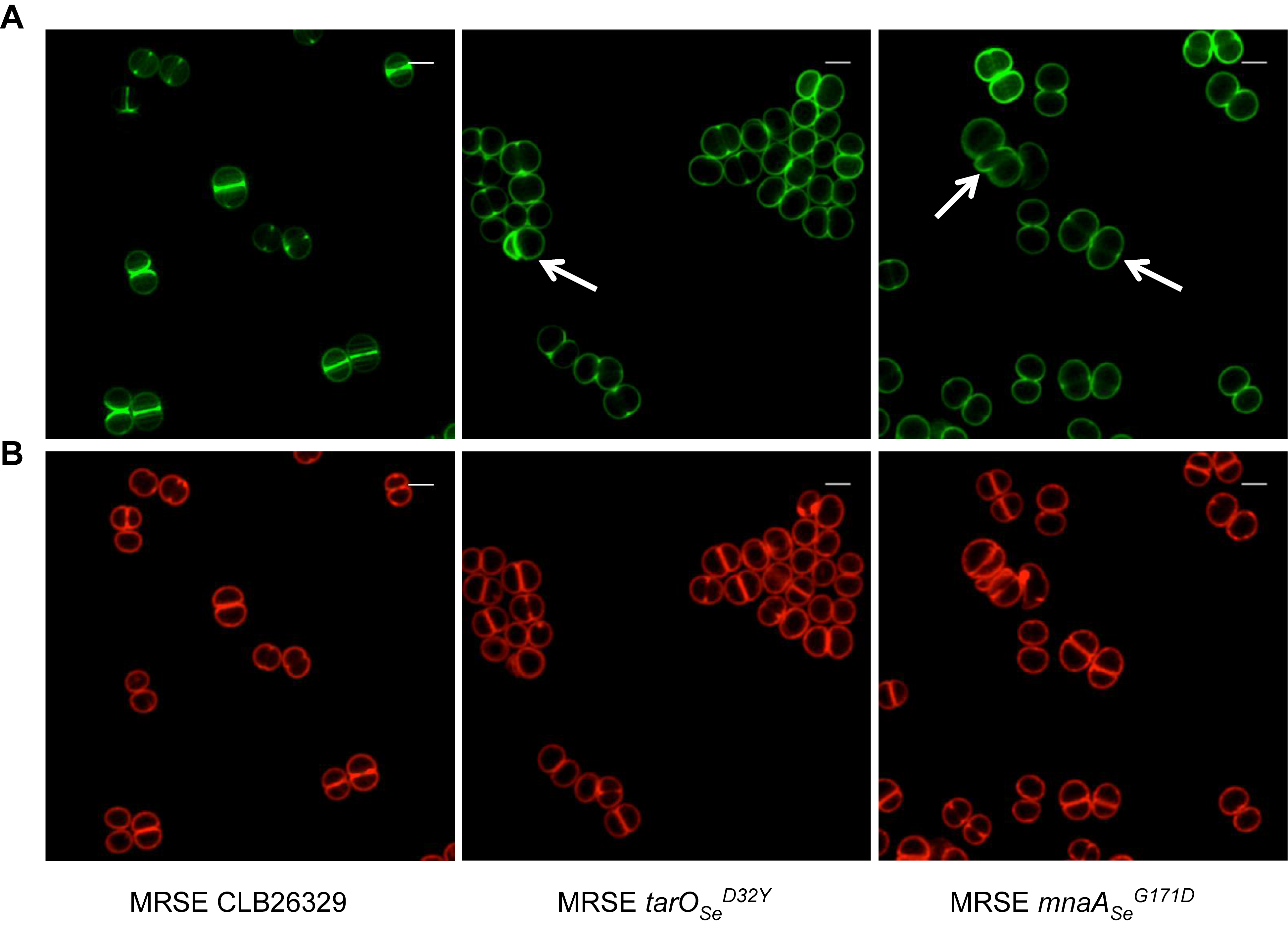

Supplement: S6 Fig — Structured illumination microscopy images of cells incubated with (A) Van-FL, and (B) Nile Red to label the cell wall and membrane, respectively. Mutants lacking WTA show increased cell size heterogeneity and septum placement defects (arrows). Scale bars represent 1 μm. (TIF) [file ppat.1005585.s007.tif]

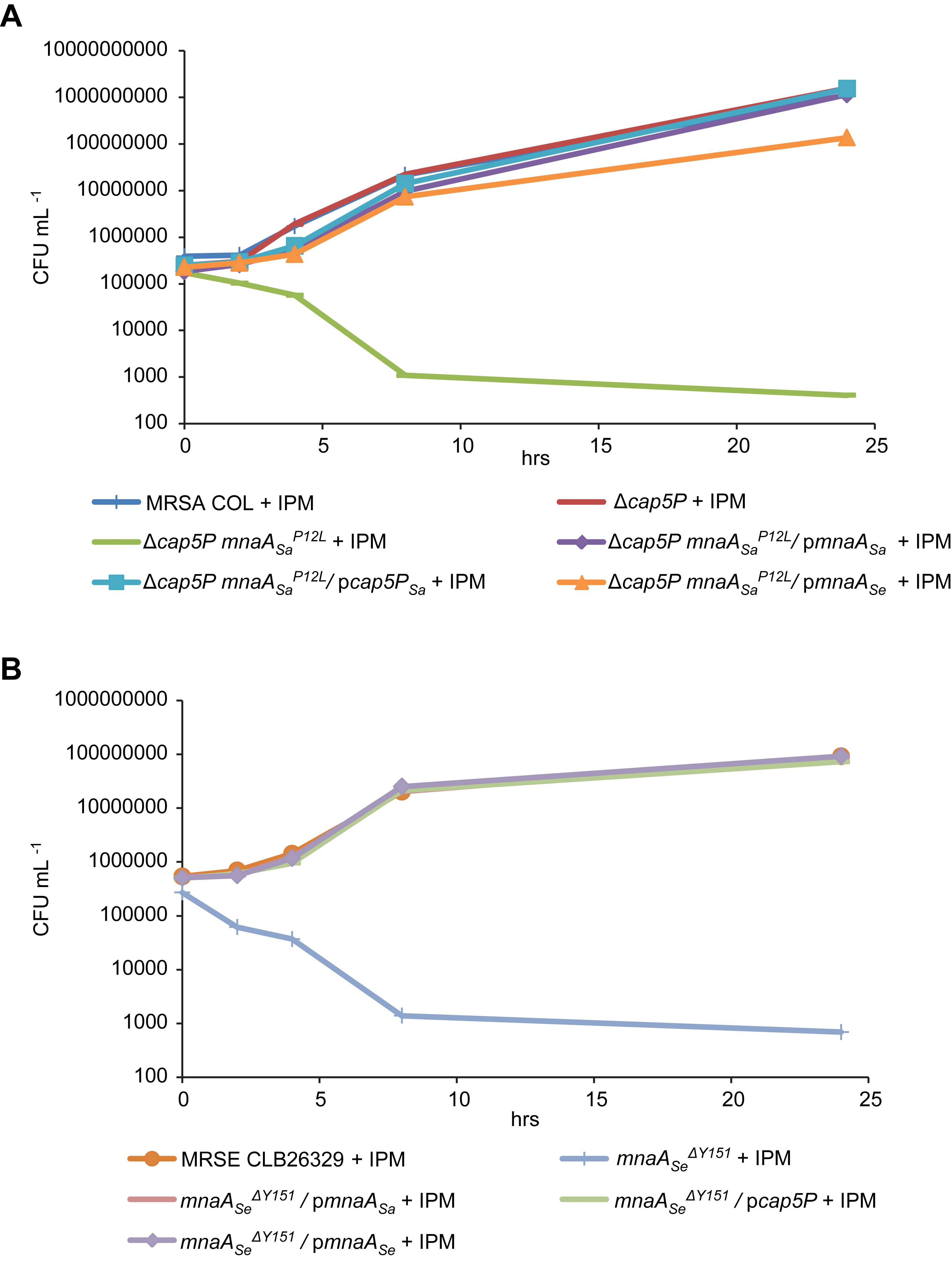

Supplement: S7 Fig — All strains were grown in the presence of 4 μg ml-1 imipenem (IPM) and CFUs measured over 24 hours. (A) MRSA COL and Δcap5P are unaffected by imipenem (4 μg ml-1), while the Δcap5P mnaA Sa P12L mutant displays a 3 log reduction in cell viability within 7 hr of imipenem treatment. Plasmid-based copies of wild-type cap5P (pcap5P), mnaA Sa (pmnaA Sa), and mnaA Se (pmnaA Se) restore resistance to imipenem. (B) Kill curves of MRSE CLB26329 performed as in (A) using mnaA Se ΔY151 and 4 μg ml-1 imipenem plasmid-based copies of wild-type cap5P (pcap5P), mnaA Sa (pmnaA Sa), and mnaA Se (pmnaA Se) fully restore resistance to imipenem. (TIF) [file ppat.1005585.s008.tif]

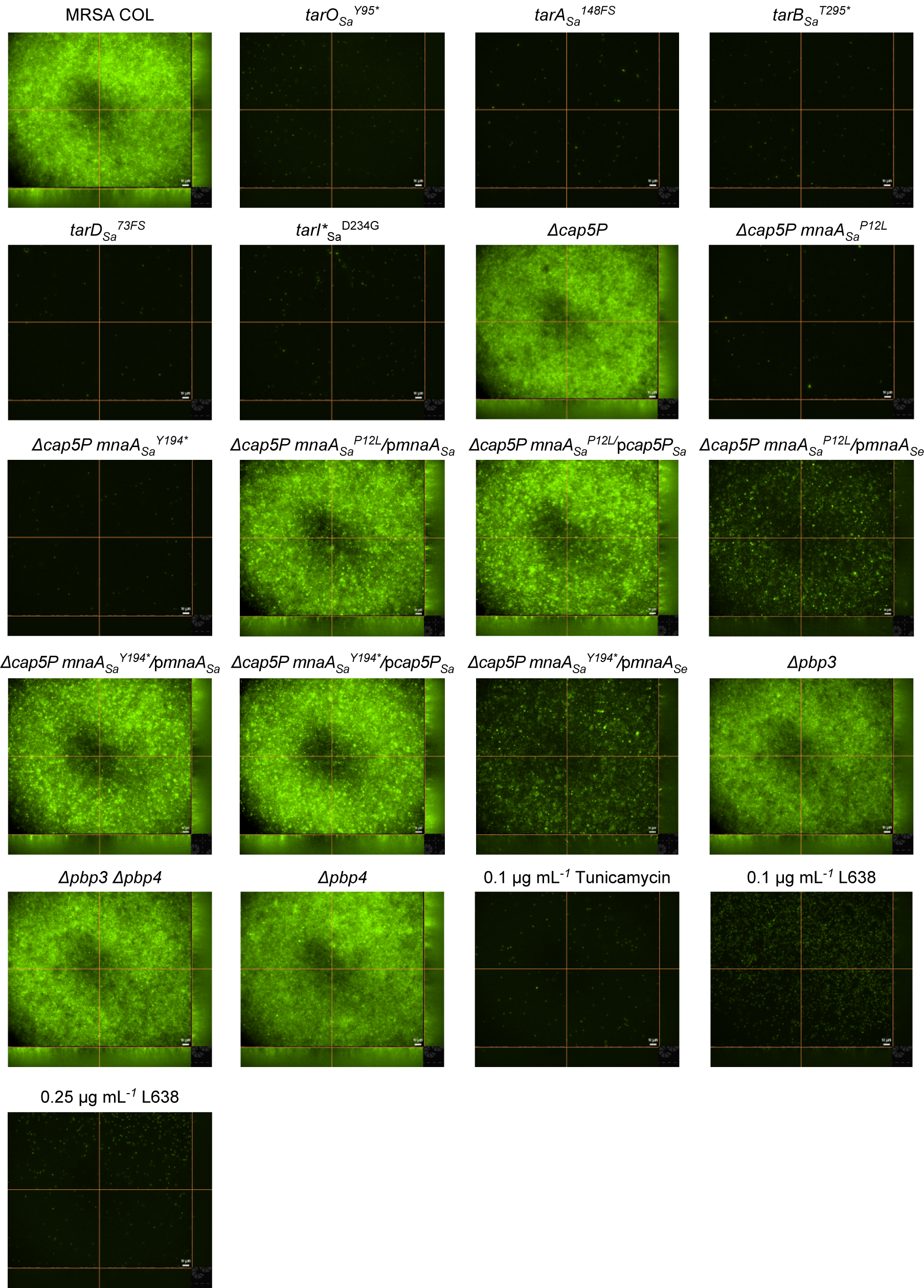

Supplement: S8 Fig — MRSA COL biofilms were grown in triplicates for 24 hours in 96-well black clear bottom plates with or without indicated sub-MIC concentrations of WTA inhibitors. Isolation and characterization of MRSA COL tarO, tarA, tarB, tarD, and tarI LOF mutants were described previously [34] and assayed here to broaden conclusions concerning the requirement of WTA in biofilm formation. Δpbp3, Δpbp4, and Δpbp3, Δpbp4 double mutants are included as negative controls for the biofilm assay. Tunicamycin and L638 treatments were performed at the indicated sub-MIC drug concentrations. Genetic complementation of described mutants was performed using plasmid-based copies of wild-type cap5P (pcap5P), mnaA Sa (pmnaA Sa), and mnaA Se (pmnaA Se) as indicated. Biofilms were stained with BacLight Green fluorescent stain. Z-stacks were obtained at 60x magnification. Scale bar = 10 μm. (TIF) [file ppat.1005585.s009.tif]

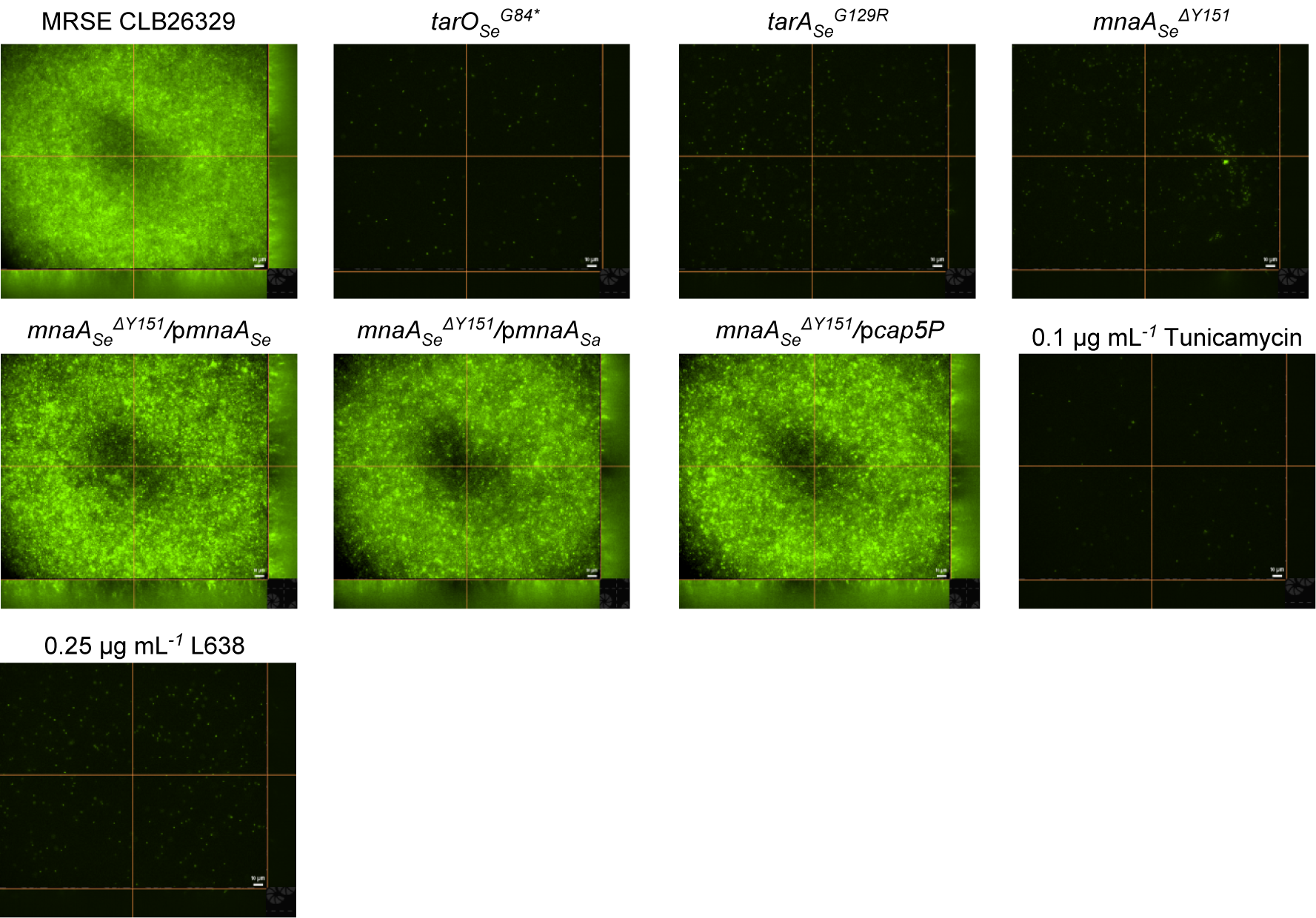

Supplement: S9 Fig — MRSE CLB26329 biofilms were grown in triplicates for 24 hours in 96-well black clear bottom plates with or without indicated sub-MIC concentrations of WTA inhibitors. Isolation and characterization of MRSE tarO, and tarA LOF mutants were described previously [34] and assayed here to broaden conclusions concerning the requirement of WTA in biofilm formation. Tunicamycin and L638 treatments were performed at the indicated sub-MIC drug concentrations. Genetic complementation of described mutants was performed using plasmid-based copies of wild-type cap5P (pcap5P), mnaA Sa (pmnaA Sa), and mnaA Se (pmnaA Se) as indicated. Biofilms were stained with BacLight Green fluorescent stain. Z-stacks were obtained at 60x magnification. Scale bar = 10 μm. (TIF) [file ppat.1005585.s010.tif]

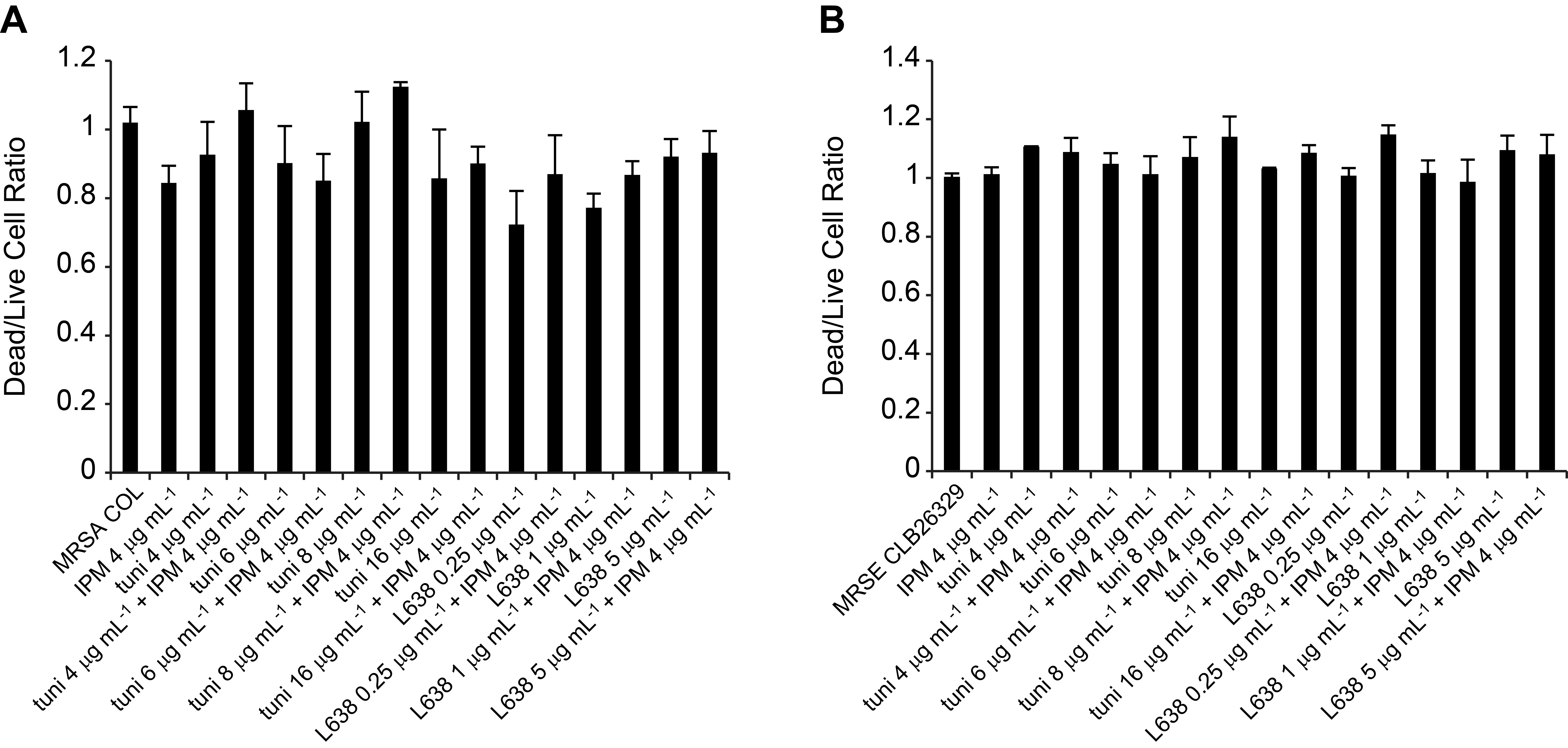

Supplement: S10 Fig — To assess biofilm killing by WTA inhibitors alone or in combination with imipenem at its clinical breakpoint, MRSA COL (A) and MRSE CLB26329 (B) biofilms were grown for 24 hours in 96-well plates before addition of WTA inhibitors tunicamycin (tuni), L638, in the absence or presence of imipenem (IPM), and then incubated for an additional 24 hours. Note drug concentrations of each inhibitor used are shown in parentheses as μg/ml. Biofilms were stained with Syto 10 for total cell staining and DEAD Red for membrane-damaged cells. Y-axis measures dead/live cell ratio. Error bars are standard deviations from triplicate experiments. No effect of any compounds tested alone or in combination was observed. (TIF) [file ppat.1005585.s011.tif]

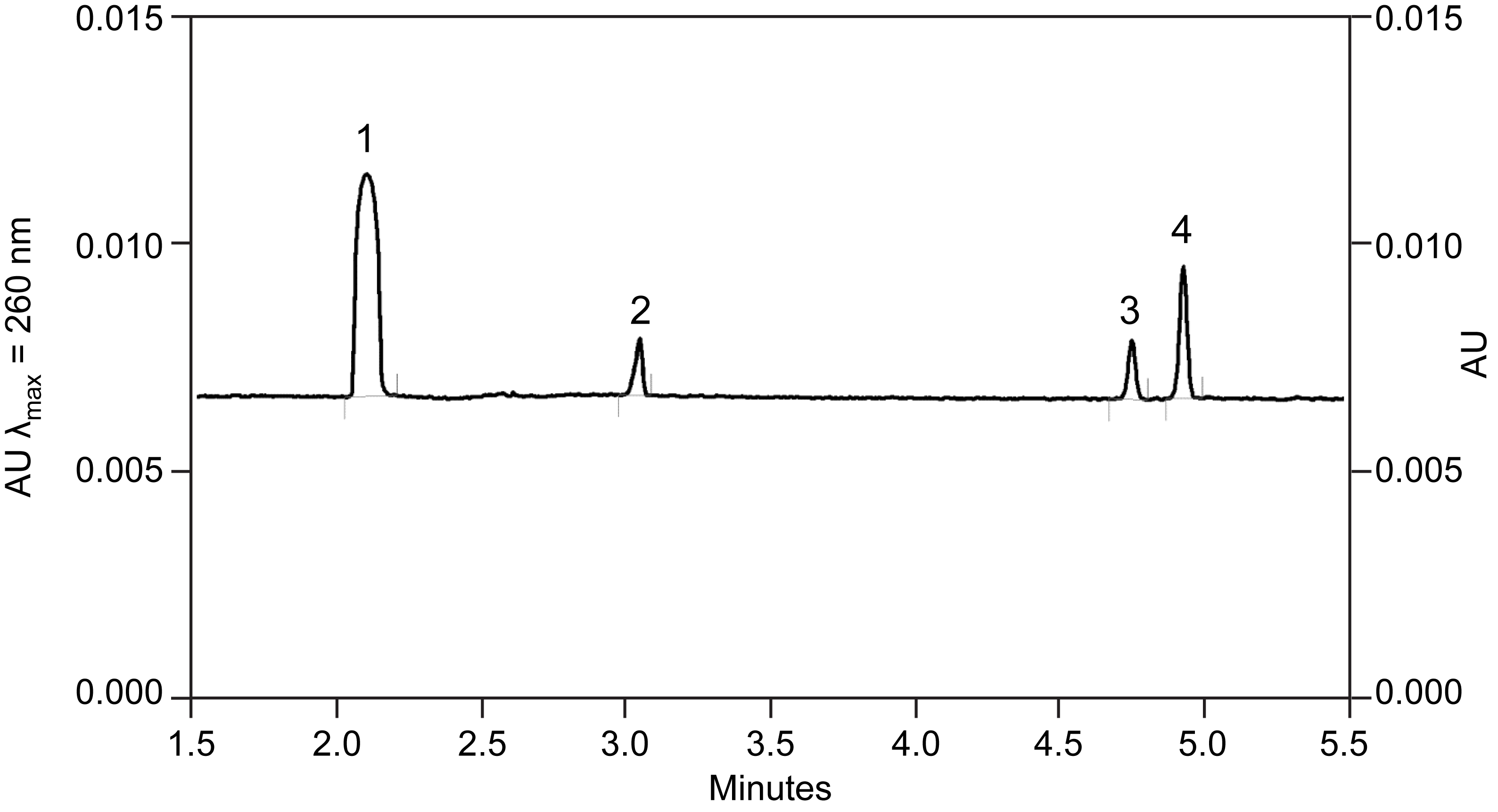

Supplement: S11 Fig — Peaks, λmax = 260 nm: 1 buffer; 2 internal standard (I.S.) adenosine; 3 UDP-ManNAc; 4 UDP-GlcNAc. (TIF) [file ppat.1005585.s012.tif]

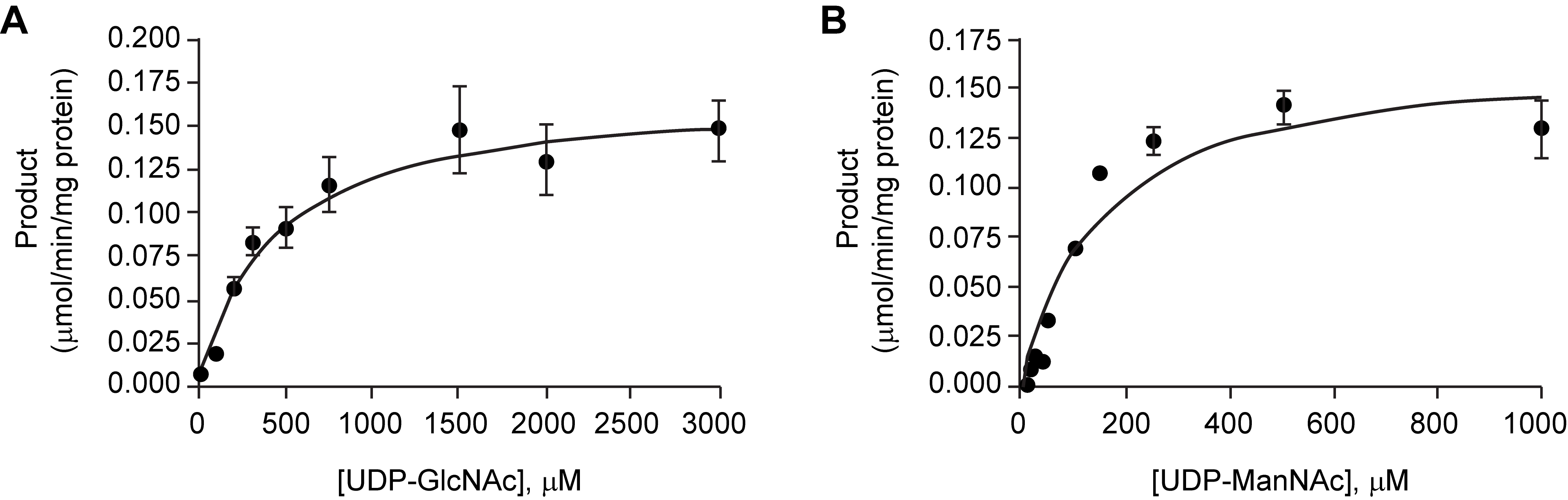

Supplement: S12 Fig — (A) Michaelis-Menten plot for MnaA forward reaction, Km for UDP-GlcNAc: 411 ± 57 μM, Vmax: 0.171 ± 0.037 μmol/min/mg protein; (B) Michaelis-Menten plot for MnaA reverse reaction, Km: 131 ± 21 μM, Vmax: 0.159 ± 0.021 μmol/min/mg protein. (TIF) [file ppat.1005585.s013.tif]

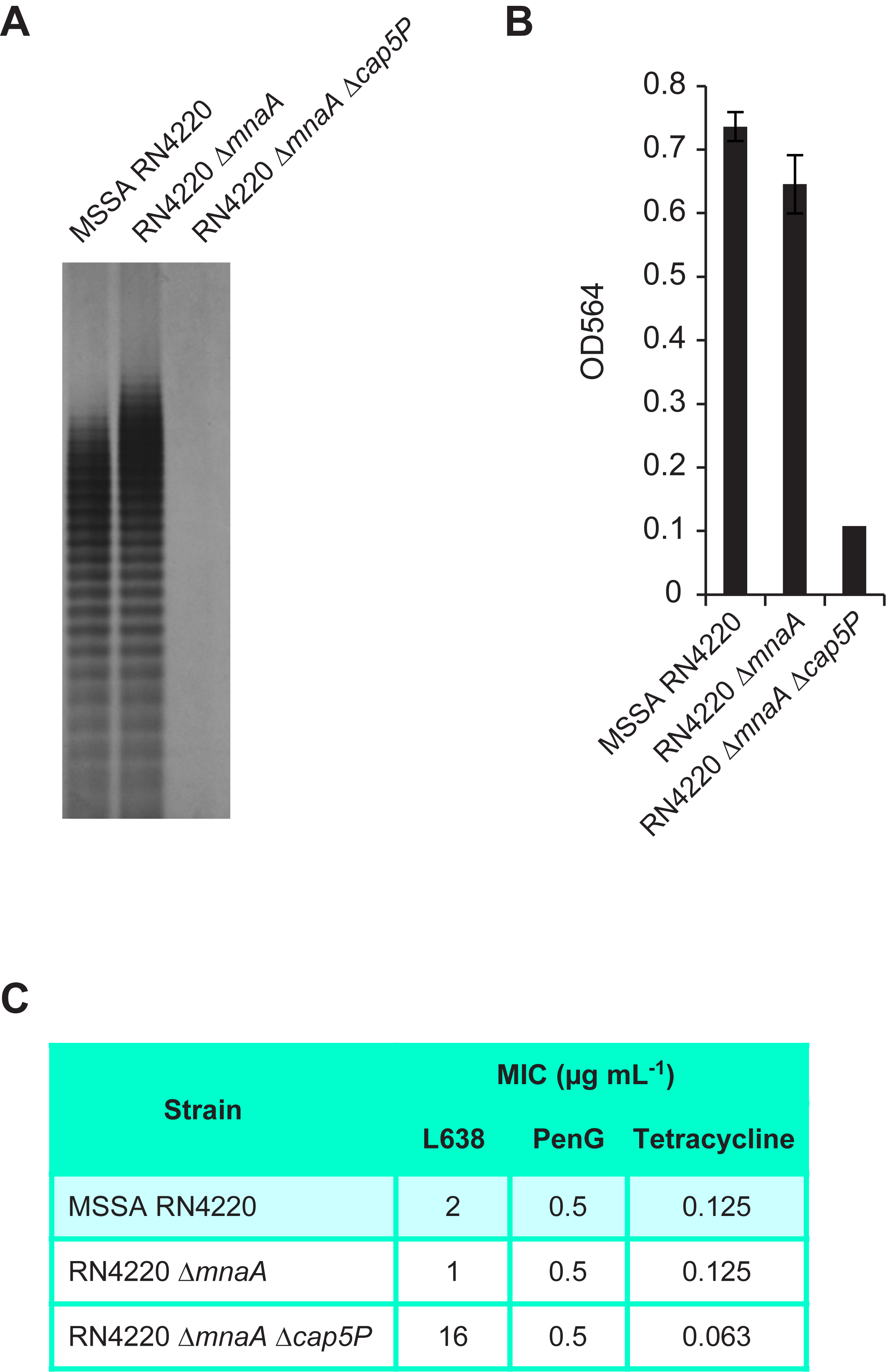

Supplement: S13 Fig — (A) WTA extraction and SDS PAGE analysis from MSSA RN4220 ΔmnaA and ΔmnaA Δcap5P. Note, wild-type MSSA RN4220 WTA polymers appear as a ladder of discretely sized. WTA material was normalized to cell biomass prior to loading. Deletion of mnaA alone does not affect WTA levels, whereas the double deletion of mnaA and cap5P does. (B) For total biofilm quantification, biofilms were grown in triplicates for 24 hours in 96-well plates. Biofilms were stained with safranin and dissolved in glacial acetic acid before OD564 was measured. Bars represent mean OD, error bars represent standard deviation. Note that deletion of mnaA alone does not affect biofilm, whereas the double deletion of mnaA and cap5P does (C) MIC values of penicillin G, tetracycline, and L638. Note that deletion of mnaA alone does not confer resistance to TarG inhibitor L638, whereas the double deletion of mnaA and cap5P does. (TIF) [file ppat.1005585.s014.tif]
